# The neuroprotective N-terminal amyloid-β core hexapeptide reverses reactive gliosis and gliotoxicity in Alzheimer’s disease pathology models

**DOI:** 10.1186/s12974-023-02807-9

**Published:** 2023-05-27

**Authors:** Megan J. Lantz, Alyssa M. Roberts, Donovan D. Delgado, Robert A. Nichols

**Affiliations:** Department of Cell and Molecular Biology, University of Hawai’i at Mānoa, Honolulu, HI USA

**Keywords:** Neuroinflammation, Alzheimer’s disease, Amyloid-β, Protective peptides, Gliosis

## Abstract

**Background:**

Alzheimer’s disease (AD) is a progressive neurodegenerative disorder characterized by accumulation of extracellular amyloid beta (Aβ) and intracellular neurofibrillary tangles, leading to chronic activation of astrocytes and microglia and persistent neuroinflammation. Aβ-linked activation of microglia and astrocytes leads to increased intracellular calcium and production of proinflammatory cytokines, impacting the progression of neurodegeneration. An N-terminal Aβ fragment (Aβ_1–15_) and a shorter hexapeptide core sequence within the N-Aβ fragment (N-Aβcore: Aβ_10–15_) have previously been shown to protect against Aβ-induced mitochondrial dysfunction, oxidative stress and apoptosis in neurons and rescue synaptic and spatial memory deficits in an APP/PSEN1 mouse model. Here, we hypothesized that the N-Aβ fragment and N-Aβcore are protective against Aβ-induced gliotoxicity, promoting a neuroprotective environment and potentially alleviating the characteristically persistent neuroinflammation present in AD.

**Methods:**

We treated ex vivo organotypic brain slice cultures from an aged familial AD mouse model, 5xFAD, with the N-Aβcore and used immunocytochemistry to assess the impact on astrogliosis and microgliosis and alterations in synaptophysin-positive puncta engulfed by microglia. Isolated neuron/glia cultures, mixed glial cultures or a microglial cell line were treated with oligomeric human Aβ at concentrations mimicking the pathogenic concentrations (μM) observed in AD in the absence or presence of the non-toxic N-terminal Aβ fragments. Resultant changes in synaptic density, gliosis, oxidative stress, mitochondrial dysfunction, apoptosis, and the expression and release of proinflammatory markers were then determined.

**Results:**

We demonstrate that the N-terminal Aβ fragments mitigated the phenotypic switch leading to astrogliosis and microgliosis induced by pathological concentrations of Aβ in mixed glial cultures and organotypic brain slice cultures from the transgenic 5xFAD mouse model, while protecting against Aβ-induced oxidative stress, mitochondrial dysfunction and apoptosis in isolated astrocytes and microglia. Moreover, the addition of the N-Aβcore attenuated the expression and release of proinflammatory mediators in microglial cells activated by Aβ and rescued microglia-mediated loss of synaptic elements induced by pathological levels of Aβ.

**Conclusions:**

Together, these findings indicate the protective functions of the N-terminal Aβ fragments extend to reactive gliosis and gliotoxicity induced by Aβ, by preventing or reversing glial reactive states indicative of neuroinflammation and synaptic loss central to AD pathogenesis.

**Supplementary Information:**

The online version contains supplementary material available at 10.1186/s12974-023-02807-9.

## Background

Alzheimer’s disease (AD) is characterized by the formation of extracellular amyloid beta (Aβ) plaques and intracellular neurofibrillary tangles comprising hyperphosphorylated tau [1, 2]. Elevation in Aβ in brain regions important for cognitive function as the result of aberrant formation and/or the dysfunctional clearance of Aβ, leads to histopathological deposition during the prodromic phase of AD [3, 4]. The elevated levels of Aβ are hypothesized to trigger multiple mechanisms including the hyperphosphorylation of tau [5–7], initiating a pathological cascade resulting in synaptic dysregulation and loss, gliosis, neuroinflammation, global calcium dysregulation, mitochondrial dysfunction and selective neuronal death leading to gross brain atrophy over time [4, 8, 9].

The accumulation of Aβ has been shown to induce significant increases in intracellular calcium concentrations ([Ca^2+^]_i_) in astrocytes and in microglia [10, 11], resulting in the release of gliotransmitters, cytokines, chemokines, complement proteins, nitric oxide (NO), and reactive oxygen species (ROS) to modulate neuronal and glial activity [12–17]. Indeed, these cells are highly responsive and undergo phenotypic shifts into reactive states, including anti- and pro-inflammatory states, in response to changing environmental cues and disease state [18–21]. Activated microglia and reactive astrocytes are known to provide many beneficial functions to slow AD disease progression including surrounding Aβ plaques and engulfing granules of non-fibrillar Aβ [22–24], forming neuroprotective glial scars [25, 26], and secreting neurotrophic factors and anti-inflammatory cytokines including IL-4, IL-10, IL-13, and TGFβ [27, 28] to promote neural stem cell differentiation and enhance long-term potentiation (LTP) [29]. However, the perpetual accumulation of Aβ in AD and the associated toxicity induced by oligomeric Aβ species skews the activation of these cells into a proinflammatory phenotype that enhances synaptic loss via microglia-mediated phagocytosis [16] and exacerbates neurodegeneration through the release of neurotoxic proinflammatory mediators, including IL-1β, IL-6 and TNFα [19, 27, 28, 30], that contribute to the development of the chronic neuroinflammation observed in AD.

Aβ species varying in length between 38 to 43 amino acids are produced by the sequential cleavage of amyloid precursor protein (APP) by β- and γ-secretases [31]. Aβ is an amphipathic molecule, containing a hydrophilic N-terminal domain that is thought to be largely unstructured [32] and a hydrophobic C-terminal domain that loops back at residues 21–23 to form an anti-parallel beta-sheet [33], which is critical to the step-wise process of generating toxic low- and high-molecular-weight soluble oligomeric Aβ species [8]. Alternatively, APP can be processed via a non-amyloidogenic pathway to produce several short N-terminal Aβ isoforms [31]. Our studies have demonstrated one of these isoforms, Aβ_1–15/16_ (which we have termed the N-Aβ fragment), and a further refined active sequence, Aβ_10–15_: YEVHHQ (referred to as the N-Aβcore) identified by structure–function analysis [34], exhibit neuroprotective functions against Aβ-induced cytotoxicity and rescue synaptic and memory deficits in APP/PSEN1 mouse models [34–36]. Here, we utilized 5xFAD transgenic mice, which overexpress human APP and presenilin 1 (PSEN1) and harbor five familial AD (FAD) mutations: Swedish (K670N, M671L), Florida (I716V), and London (V717I) APP mutations as well as two mutations in PSEN1 (M146L and L286V) [37]. In this study, we investigated the impact of the N-Aβ fragment and N-Aβcore on the activation of astrocytes and microglia, the release of proinflammatory mediators induced by Aβ, and the mitigation of enhanced synaptic engulfment by microglia utilizing four model systems: organotypic slice cultures from the 5xFAD transgenic mouse model, isolated murine cortical mixed neuron–glia cultures, isolated murine cortical mixed glial cultures, and the murine microglial BV2 cell line. Moreover, the neuroprotective potential of these N-terminal Aβ fragments against the cytotoxic oxidative stress, mitochondrial dysfunction and apoptosis of astrocytes and microglia induced by prolonged treatment with Aβ was also determined.

## Results

### N-Aβcore attenuates Aβ-induced reactive gliosis in 5xFAD ex vivo organotypic slice cultures

**Fig. 1:**
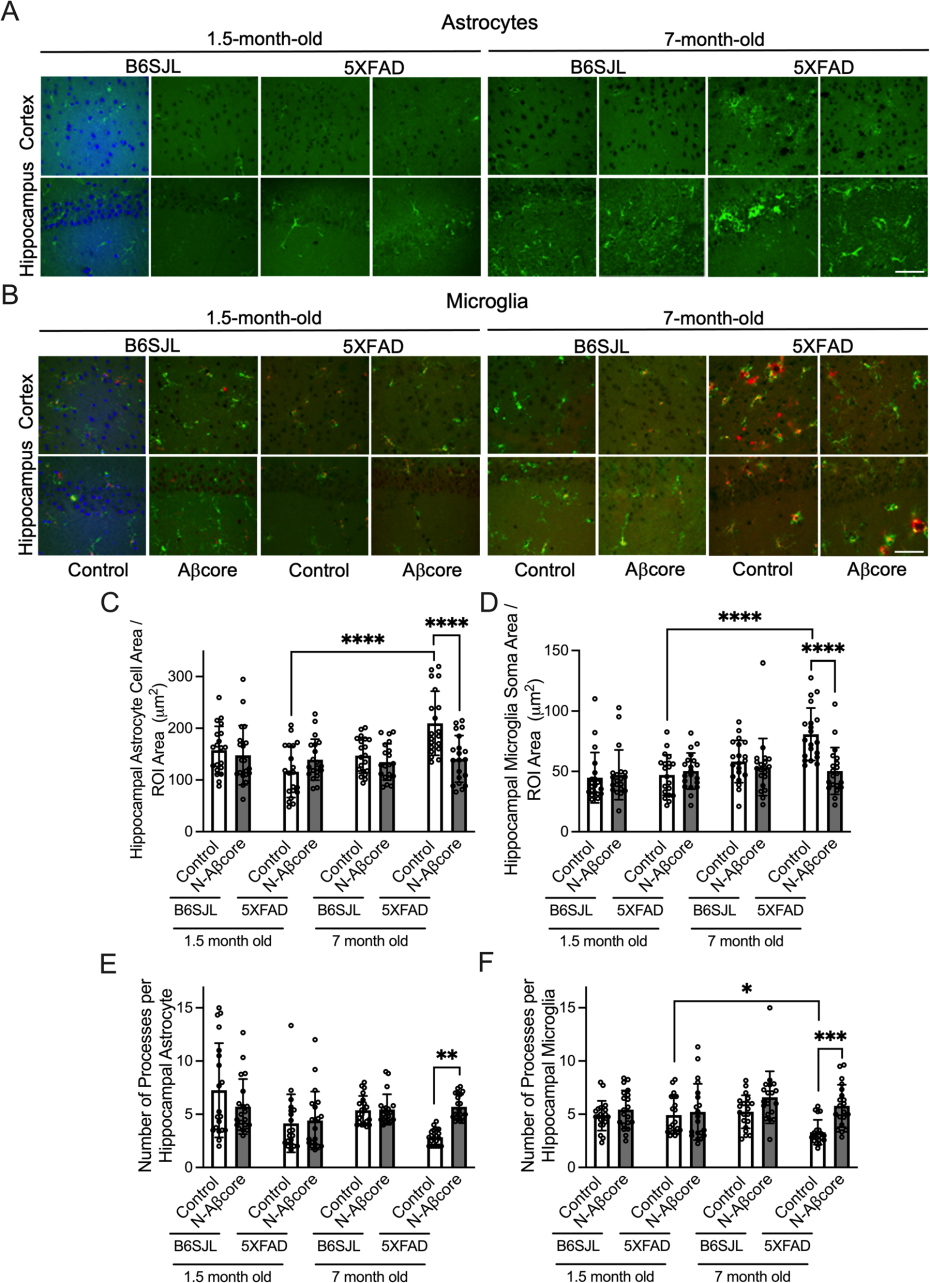
Treatment with 1 μM Aβcore reduces astrogliosis and microgliosis in organotypic slice cultures from 7-month-old 5xFAD mice. Organotypic slice cultures from 1.5- and 7-month-old B6SJL (background) and 5xFAD mice were treated daily for 7 days with media only (Control) or 1 μM N-Aβcore. **A** Representative images of GFAP-positive astrocytes (green) and **B** Iba1-positive (green) / CD68-positive (red) microglia in the somatosensory cortex (top) and CA1 region of the hippocampus (bottom) from 1.5- and 7-month-old B6SJL and 5xFAD mice. The first pairs of 1.5-month-old Control B6JSL images were counterstained with DAPI (blue). Scale bar: 50 μm. Analysis of cell area per ROI 34,000 μm.^2^ for GFAP-positive astrocytes (**C**) and Iba1-positive microglia (**D**) (*n* = 20 cells across 6 slices from 3 mice for each condition). Number of processes per astrocyte (**E**) and microglia (**F**) (*n* = 20 cells across 6 slices from 3 mice per condition). Data are means ± SD. All data in **C**–**E** analyzed via two-way ANOVA with Tukey post hoc test as compared to Control for each treatment day. *p < 0.05; **p < 0.01; ***p < 0.001; ****p < 0.0001

To investigate the effect of the N-Aβcore on gliosis induced by Aβ in an accessible, intact ex vivo preparation, cultures of coronal brain slices from 1.5- or 7-month-old 5xFAD or wild-type B6SJL (genetic background) mice were treated daily for 7 days with media only (Control) or 1 μM N-Aβcore prior to the assessment of any morphological alterations and/or protein expression changes of several phenotype-specific cell markers via immunofluorescence staining. The 5xFAD transgenic mouse model was used as it recapitulates several major AD pathological features [37]. The ages for the model were chosen as Aβ rises at 1.5 months of age in 5xFAD mice without the development of gliosis or plaques, while 7-month-old 5xFAD mice display robust synaptic and behavioral deficits as well as severe gliosis and neuron loss [37]. The level of expression of the astrocyte-specific protein, GFAP, was utilized as a marker for activated astrocytes [25], denoting upregulation of GFAP as a fluorescence intensity value one standard deviation or more (> 1 SD) above the mean GFAP expression in the untreated control samples (referred to as GFAP^High^ expressing astrocytes). Similarly, expression of the microglial markers Iba1 (a microglia-specific calcium-binding protein) and CD68 (also known as LAMP4, a microglial lysosomal and endosomal-associated protein) increases as microglia transition to an activated state [38, 39]. In agreement with Oakley et al*.* [37], astrogliosis and microgliosis were not observed in 5xFAD slices at 1.5 months of age (Fig. 1; Additional file 1: Appendix, Fig. S1), though small trends were present in phenotypic measures (GFAP expression, cell size or process number) for astrocytes (Fig. 1C, E; Additional file 1: Appendix, Fig. S1A, B; Table 1), but not microglia (Fig. 1D, F; Additional file 1: Appendix, Fig. S1C, D; Table 1), in two vulnerable brain regions, the cortex and hippocampus, from 5xFAD slices as compared to matched slices from B6SJL mice. Moreover, treatment with N-Aβcore did not significantly alter any of the phenotypic measurements utilized to study gliosis in astrocytes or microglia, except for a significant decrease in the number of GFAP^High^ expressing cortical astrocytes in 1.5-month-old 5xFAD slices compared to the B6SJL control slices (Additional file 1: Appendix, Fig. S1A).

**Table 1:** Statistical comparison of glial marker phenotypes in organotypic slices from 1.5-month-old and 7-month-old wild-type (B6SJL) and 5xFAD mice under various treatments (Fig. 1)

Treatment	Hippocampus		Cortex	
	Control	N-Aβcore	Control	N-Aβcore
Microglia: Iba1^High^ CD68^High^				
1.5 mo vs 7 mo B6SJL	*p* < 0.0001	*p* < 0.0001	*p* = 0.0002	*p* = 0.0016
1.5 mo vs 7 mo 5xFAD	*p* < 0.0001	*p* < 0.0001	*p* < 0.0001	*p* < 0.0001
1.5 mo B6SJL Control vs N-Aβcore		*p* = 0.9996		*p* = 0.7463
1.5 mo 5xFAD Control vs N-Aβcore		*p* = 0.4282		*p* = 0.7463
7 mo B6SJL Control vs N-Aβcore		*p* = 0.0296		*p* = 0.3023
7 mo 5xFAD Control vs N-Aβcore		*p* = 0.1138		*p* = 0.4216
Astrocytes: GFAP^High^				
1.5 mo vs 7 mo B6SJL	*p* = 0.9632	*p* = 0.9996	*p* = 0.0946	*p* = 0.9997
1.5 mo vs 7 mo 5xFAD	*p* = 0.0003	*p* = 0.0124	*p* < 0.0001	*p* = 0.0620
1.5 mo B6SJL Control vs N-Aβcore		*p* = 0.9513		*p* = 0.9997
1.5 mo 5xFAD Control vs N-Aβcore		*p* = 0.9940		*p* = 0.0339
7 mo B6SJL Control vs N-Aβcore		*p* = 0.3343		*p* = 0.1082
7 mo 5xFAD Control vs N-Aβcore		*p* = 0.3853		*p* < 0.0001

*p* values from Tukey post hoc tests of two-way ANOVAs of number of hippocampal and cortical cells positive for the phenotypic markers

The number of reactive astrocytes did not significantly change with age in slices from the B6SJL mouse line (Fig. 1A, C, E; Additional file 1: Appendix, Fig. S1A, B), nor was there any effect with 1 μM N-Aβcore treatment. These data indicate a stable astrocyte phenotypic morphology out to 7 months of age in B6SJL mice. Similarly, no significant alteration in microglial morphology was observed in aged B6SJL slice cultures (Fig. 1B, D, F), though there was an increase in the number of Iba1^High^/CD68^High^ expressing microglia in slice cultures from 7-month-old compared to 1.5-month-old mice (Additional file 1: Appendix, Fig. S1C, D). Unlike the B6SJL slice cultures, a dramatic increase in astrogliosis and microgliosis was observed in media only treated (Control) aged 5xFAD slice cultures (Fig. 1; Additional file 1: Appendix, Fig S1), which was partially rescued by daily treatment for 7 days with 1 μM N-Aβcore. N-Aβcore treatment did mitigate the increase in astrocyte and microglial cell and soma area, respectively (Fig. 1C, D), and the decrease in astrocytic and microglial process number (Fig. 1E, F) in slice cultures from the 7-month-old mice. Moreover, treatment with N-Aβcore substantially reduced the number of GFAP^High^ expressing cortical astrocytes in aged 5xFAD slices (Fig. 1*A*; Additional file 1: Appendix, Fig S1A). However, N-Aβcore treatment did not significantly affect the number of hippocampal GFAP^High^ expressing astrocytes (Additional file 1: Appendix, Fig. S1B) nor cortical or hippocampal Iba1^High^ CD68^High^ expressing microglia in 7-month-old 5xFAD slices (Fig. 1B; Additional file 1: Appendix, Fig. S1C, D), perhaps due to the relatively short treatment timeframe. In contrast, the neuronal population, notably reduced in aged 5xFAD [37], was increased in the cortical and hippocampal regions of 5xFAD slice cultures on treatment with the N-Aβcore (Additional file 1: Fig. S2), indicating reversal of this deficit. Together, these data suggest that a short treatment with N-Aβcore can reverse the morphology of reactive astrocytes and microglia in the presence of pathological Aβ accumulation into a morphology more typical of ‘resting’ astrocytes and microglia, in parallel to reversal by N-Aβcore of neuron loss in the same ex vivo familial AD model.

### N-Aβ fragment and N-Aβcore mitigate reactive astrogliosis and microgliosis induced by Aβ in vitro

Aβ is a naturally occurring molecule in the brain under homeostatic conditions [3], with potential neuromodulatory activity at picomolar concentrations [34, 40, 41] and toxic properties at higher concentrations [9, 42–44]. Yet, its action on glial cells at various, and especially lower, concentrations is not well understood. To determine whether lower concentrations (pM–nM) are sufficient to evoke changes in glial intracellular calcium responses and to study whether the N-Aβ fragments elicit differential calcium responses compared to Aβ, primary mixed glia cultures were used to measure the induced calcium responses in morphologically identified astrocytes and microglia during perfusion with either Aβ_1-42_, N-Aβ fragment or N-Aβcore at increasing concentrations (100 pM, 100 nM, 1 μM or 2.5 μM) in live-cell imaging (Additional file 1: Appendix, Fig. S3A, B). Application of all Aβ peptides tested elicited similar weak, sustained calcium responses at 100 pM (Additional file 1: Appendix, Fig. S3A, B). A similar weak, but sustained, calcium response with the application of 100 nM Aβ_1–42_, no response with 100 nM N-Aβ fragment and an attenuated response with 100 nM N-Aβcore was observed. These findings are in sharp contrast to that found for regulation of [Ca^2+^]_i_ in neurons and presynaptic nerve terminals, where 100 pM–1 nM stimulation by Aβ_1–42_ triggers maximum calcium responses [34], and thus indicate that glia are decidedly lower in sensitivity to Aβ as compared to neurons.

On the other hand, increasing the concentration of Aβ_1–42_ to pathological levels (μM) induced notable prolonged, robust calcium responses, especially with acute 2.5 μM treatment, (Additional file 1: Appendix, Fig. S3A, B). Interestingly, application of 1 μM N-Aβ fragment or N-Aβcore evoked an attenuated response compared to Aβ_1–42_ at the same concentration and a transient or no response, respectively, at the highest concentration tested, 2.5 μM (Additional file 1: Appendix, Fig. S3A, B). Importantly, the removal of Ca^2+^ ions from the media attenuated the calcium responses induced by application of 1 μM Aβ_1–42_, in agreement with the findings of other studies [45–47], 1 μM N-Aβ fragment or 1 μM N-Aβcore (Additional file 1: Appendix, Fig. S3C), suggesting that the various Aβ peptide-induced increases in [Ca^2+^]_i_ were triggered by the influx of extracellular Ca^2+^ ions.

The observation of attenuated calcium responses elicited by 1 μM concentrations of the N-Aβ fragments (Additional file 1: Appendix, Fig. S3A, B) prompted an investigation into whether the N-Aβ fragment or N-Aβcore could mitigate the robust calcium response induced by 2.5 μM Aβ_1–42_ in astrocytes and microglia. To address this question, we measured the [Ca^2+^]_i_ induced by live-cell co-perfusion with 2.5 μM Aβ_1–42_ + 1 μM N-Aβ fragment or 1 μM N-Aβcore compared to 2.5 μM Aβ_1–42_ alone in morphologically identified astrocytes and microglia in mixed glia cultures. In agreement with our previous results using a hybrid neuroblastoma cell line [35], the N-Aβ fragment completely abolished and the N-Aβcore attenuated the robust, prolonged intracellular calcium response induced by perfusion with 2.5 μM Aβ_1–42_ (Additional file 1: Appendix, Fig. S3D). All together, these results suggest the N-Aβ fragments differentially regulate intracellular calcium responses compared to Aβ_1–42_ and may directly attenuate the action of full-length Aβ_1–42_ at known Aβ target receptors in astrocytes and microglia.

The weak elicited calcium responses in primary astrocytes and microglia to 100 pM concentrations of all the Aβ peptides tested and the attenuated responses of the N-Aβ fragments compared to Aβ_1–42_ at 1 μM concentrations (Additional file 1: Appendix, Fig. S3A, B) prompted an investigation into whether 100 pM treatment with these Aβ peptides could induce an activation of primary astrocytes and microglia, and whether co-treatment of either of the N-Aβ fragments could mitigate the phenotypic shift and activation of these glial cells induced by Aβ_1–42_ at 1 μM concentrations. Unsurprisingly, 100 pM treatment with all Aβ peptides tested, alone or in combination, did not induce reactive astrocytes or microglia after 1–3 days of treatment (Additional file 1: Fig. S4), once again in contrast to the regulatory action of 100 pM Aβ on neuronal systems [34, 40, 41].

**Fig. 2:**
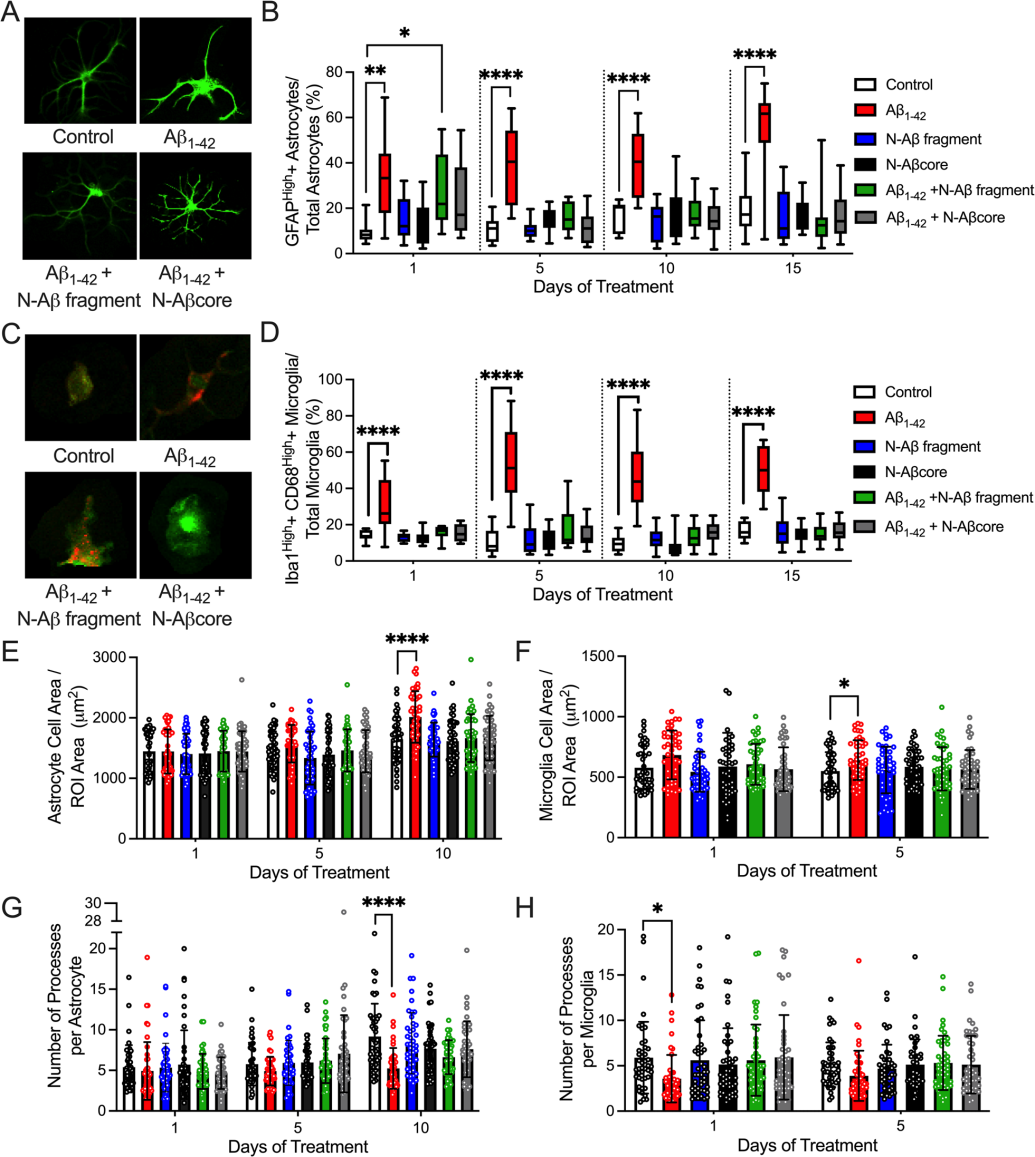
Co-treatment with the N-Aβ fragment or N-Aβcore mitigates the phenotypic shift in primary cortical astrocytes and microglia induced by Aβ_1–42_ in mixed glia cultures. **A** Representative images of GFAP-positive astrocytes after daily treatment for 5 days with media only (Control), 1 μM Aβ_1–42_, 1 μM Aβ_1–42_ + 1 μM N-Aβ fragment or 1 μM Aβ_1–42_ + 1 μM N-Aβcore. Scale bar: 100 μm. **B** The percentage of astrocytes with a relatively high level of GFAP expression after 1, 5, 10 or 15 days of treatment with media only (Control), 1 μM Aβ_1–42_, 1 μM N-Aβ fragment, 1 μM N-Aβcore, 1 μM Aβ_1–42_ + 1 μM N-Aβ fragment, or 1 μM Aβ_1–42_ + 1 μM N-Aβcore. (Threshold for GFAP^high^: 1 SD over mean intensity value.) **C** Representative images of Iba1(green)/CD68(red)-positive microglia after daily treatment for 5 days with media only (Control), 1 μM Aβ_1–42_, 1 μM Aβ_1–42_ + 1 μM N-Aβ fragment or 1 μM Aβ_1–42_ + 1 μM N-Aβcore. Scale bar: 100 μm. **D** The percentage of microglia with a relatively high level of Iba1 and CD68 expression after 1, 5, 10 or 15 days of treatment as described in **B**. (Threshold for Iba1^high^ and CD68 ^high^: 1SD over mean intensity values). Analysis of astrocyte (**E**) and microglia (**F**) cell area per field of view (ROI, 34,000 μm^2^) after treatment for 1, 5 or 10 days with the treatment solutions described in **B** (*n* = 45 cells per treatment condition). Number of processes per astrocyte (**G**) or microglia (**H**) as determined by Sholl analysis after treatment with solutions described in **B**. for 1, 5 or 10 days. (*n* = 45 cells per treatment condition). Images in **A** and **C** obtained on a Leica TCS SP8 confocal microscope. Data are means ± SD. All data in **B** and **D**–**H** analyzed via two-way ANOVA with Tukey post hoc test as compared to Control for each treatment day. *p < 0.05; **p < 0.01; ***p < 0.001; ****p < 0.0001

Numerous studies indicate pathological (high nM to μM) concentrations of Aβ_1–42_ trigger a phenotypic shift in astrocytes and microglia from a ‘resting’ state to an activated state [16, 21, 48, 49]. In agreement, Aβ_1–42_ treatment activated primary astrocytes and microglia as evidenced by the increase in GFAP or Iba1 and CD68 expression, respectively, on each day tested (Fig. 2A–D; Additional file 1: Appendix, Fig. S5) as well as the appearance of hypertrophic astrocytes (Fig. 2A, E, G; Additional file 1: Appendix, Fig S5A) and amoeboid-like microglia (Fig. 2C, F, H; Additional file 1: Appendix, Fig. S5B). In agreement with the abolishment or attenuation of Aβ_1–42_-elicited calcium responses by the N-Aβ fragment and N-Aβcore, respectively (Additional file 1: Appendix, Fig. S3D), co-treatment with the N-Aβ fragments mitigated the appearance of Aβ_1–42_-induced reactive astrocytes and microglia (Fig. 2B, D–G; Additional file 1: Appendix, Fig. S5). Importantly, neither treatment with the N-Aβ fragment nor the N-Aβcore alone induced the activation of primary astrocytes or microglia on any of the treatment days tested (Fig. 2; Additional file 1: Appendix, Fig. S5), in agreement with the attenuated calcium responses elicited by these N-Aβ fragments compared to Aβ_1–42_ (Additional file 1: Appendix, Fig. S3A, B). All together, these results suggest the N-Aβ fragment and N-Aβcore sequences do not induce reactive astrocytes or microglia alone and are able to attenuate the activation of these cells by Aβ_1–42_.

### N-Aβcore reduced levels of Aβ-induced proinflammatory mediators

**Fig. 3:**
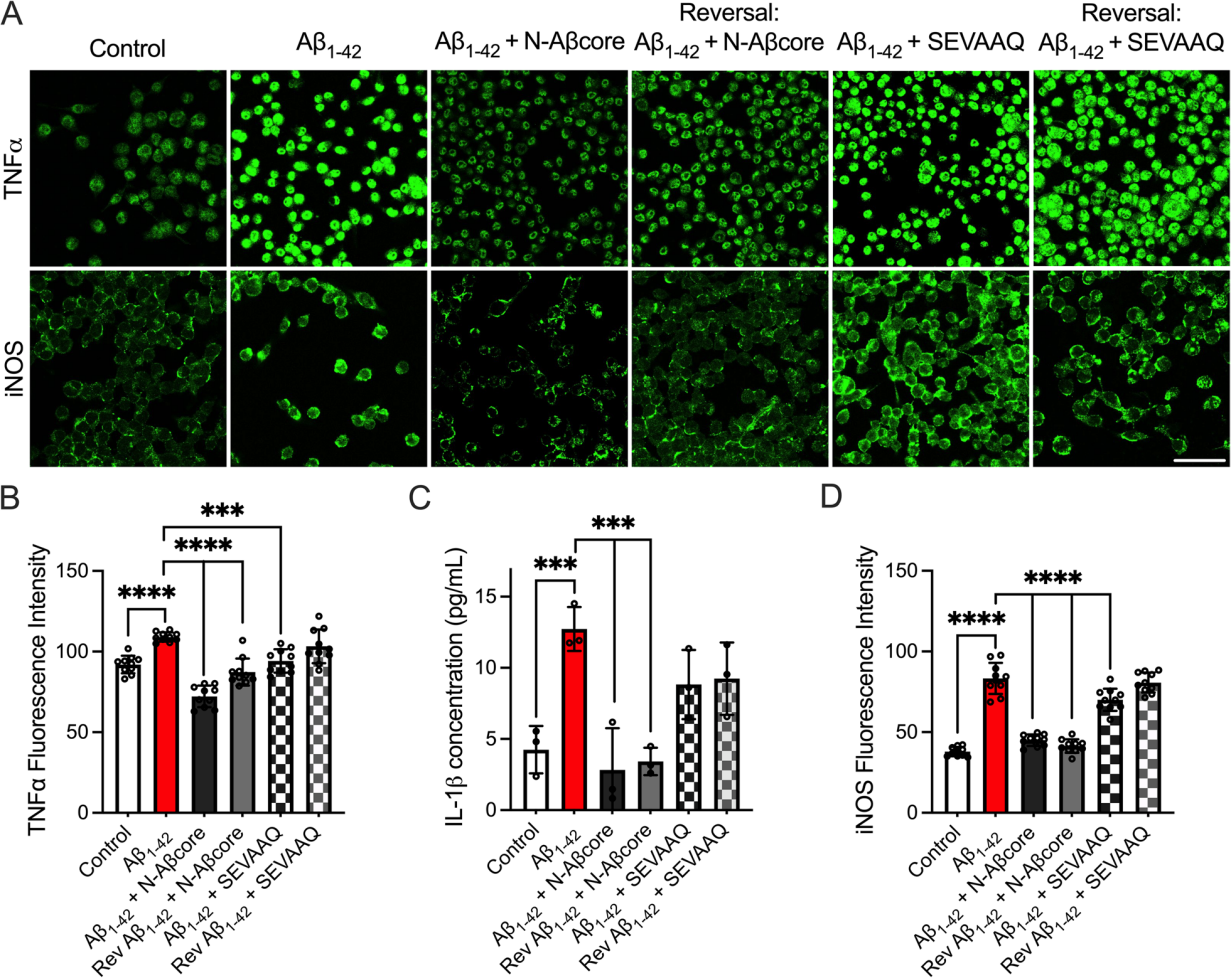
N-Aβcore co-treatment attenuates Aβ_1–42_-induced increases in proinflammatory markers in BV2 cells. BV2 cells were treated daily for 5 days with media only (Control), 500 nM Aβ_1–42_, 500 nM Aβ_1–42_ + 500 nM N-Aβcore or 500 nM Aβ_1–42_ + 500 nM SEVAAQ (inactive substituted N-Aβcore) or daily for 6 days with 500 nM Aβ_1–42_ under the reversal conditions (see Fig. 6A, legend). **A** Representative images of immunostaining for TNFα (top) and iNOS expression (bottom) in BV2 cells. Images obtained using a 63X objective on a Leica TCS SP8 confocal microscope. Scale bar: 50 μm. **B** Quantification of TNFα (*n* = 3) and **D** iNOS (*n* = 3) fluorescence intensities in A. Data shown as means ± 95% confidence intervals. **C** Quantification of IL-1β release into the BV2 cell culture media via ELISA after treatment as described above. Data shown as means ± SD. All data in **B**–**D** analyzed via one-way ANOVA with Dunnett post hoc test as compared to Aβ_1–42_. **p < 0.01; ***p < 0.001; ****p < 0.0001. *Rev* reversal condition

A significant increase in the concentration of proinflammatory cytokines including IL-1β, IL-6, IL-12, IL-18, and TNFα has been observed in the brains and cerebrospinal fluid of AD patients [50–52] and may directly contribute to synaptic degeneration [53]. Microglia are implicated as the primary mediators of inflammation in the brain [51], and likely drive much of the neuroinflammation in AD. Moreover, the inducible form of nitric oxide (NO) synthase (iNOS) is expressed by neurons and glial cells in response to proinflammatory cytokines [54] and can increase the local production of NO. NO, in turn, has been implicated in axonal and synaptic damage, the inhibition of mitochondrial respiration and the induction of neuronal apoptosis [53]. As treatment with N-Aβcore mitigated the Aβ_1–42_-induced activation of cultured microglia (Fig. 2), we next sought to investigate the impact of this N-terminal Aβ core peptide on the expression and release of proinflammatory mediators from homogeneous cultures of mouse microglia cells via immunostaining and ELISA, respectively. As obtaining high purity mouse microglia cultures presents significant challenges, the immortalized murine microglial line BV2 [55] was utilized as a homogeneous microglial culture system, which has been shown to release proinflammatory mediators after induction [56, 57], equivalent to that of primary microglia from which BV2 was derived. Treatment with Aβ_1–42_ alone significantly increased the expression of the proinflammatory mediators TNFα and iNOS in and the release of IL-1β from BV2 microglial cells (Fig. 3). For comparison, a trend toward an increase in secreted neurotrophin brain-derived neurotrophic factor (BDNF) following treatment with Aβ was also found (Additional file 1: Fig. S6). Moreover, Aβ_1–42_ treatment induced morphological changes in BV2 microglial cells indicative of activation (increased soma size and decreased the number of primary branches) as well as increased the cellular expression of the lysosomal protein CD68 (Additional file 1: Appendix, Fig. S7) compared to the untreated control. These morphological alterations, increased CD68, TNFα, and iNOS expression as well as increased IL-1β release were mitigated with co-treatment of the N-Aβcore, but not the inactive substituted N-Aβcore sequence SEVAAQ [35] (Additional file 1: Appendix, Fig. S7; Fig. 3). Surprisingly, the addition of the N-Aβcore one day after beginning daily Aβ_1–42_ treatments, under the reversal condition (see treatment schematic in Fig. 6A), yielded similar results (Additional file 1: Appendix, Fig. S7; Fig. 3), suggesting the N-Aβcore can potently both attenuate and rescue the Aβ_1–42_-induced expression and release of proinflammatory mediators from BV2 microglial cells.

### N-Aβcore attenuates Aβ-induced enhanced synaptic engulfment by reactive microglia

**Fig. 4:**
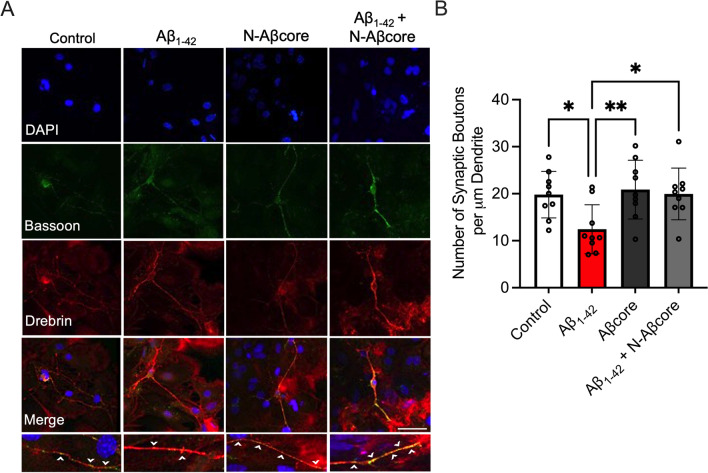
N-Aβcore attenuates synaptic loss induced by Aβ_1–42_. **A** Representative images of synapses, as determined by the colocalization of the synaptic protein Bassoon (green) and postsynaptic protein Drebrin (red), in primary cortical neurons from mixed neuronal/glia cultures after 5 days daily treatment with media only (Control), 1 μM Aβ_1–42_, 1 μM N-Aβcore, or 1 μM Aβ_1–42_ + 1 μM N-Aβcore. Bottom row: Magnified images of Bassoon-labeled presynaptic puncta (green) on Drebrin (red) labeled dendrite (white arrowhead). Images obtained on a Leica SP8 confocal microscope using a 63× objective. Scale bar: 50 μm. Primary antibodies [1:200 Mouse anti-Bassoon antibody (Abcam) and 1:200 Rabbit anti-Drebrin (Abcam)] were omitted in the secondary only sample. **B** The number of synaptic boutons per μM dendrite after treatments as described in **A**. (*n* = 9), where *n* represents the number of replicates. Data are means ± SD, analyzed via one-way ANOVA with Dunnett post hoc test as compared to 1 μM Aβ_1–42_

Of the important AD hallmarks, synaptic dysfunction and loss correlate most strongly with cognitive decline [58, 59]. Oligomeric Aβ has been shown to target synapses [60, 61], which may be later eliminated by microglia in a complement-dependent manner [16]. Previously, we have shown the N-Aβ fragment can rescue synaptic and memory deficits in aged 5xFAD mice [35] at an age when significant Aβ burden and gliosis are present [37]. Thus, we sought to determine whether the N-Aβcore could mitigate oligomeric Aβ-induced, microglia-mediated synaptic loss and thus have a longer term impact on neurodegenerative processes. Mixed neuron–glia cultures were treated with media only (Control), 1 μM Aβ_1–42_, 1 μM Aβcore or 1 μM Aβ_1–42_ + 1 μM N-Aβcore daily for 5 days prior to examining the number of synapses present via double-label immunofluorescence staining. Synaptic boutons were defined via the colocalization of the presynaptic marker Bassoon [62] and the postsynaptic marker Drebrin [63]. Similar to other studies [60, 61, 64], we found a reduction in the number of synapses after Aβ_1–42_ treatment of the cultures (*p* = 0.0209), while treatment with the N-Aβcore alone did not alter synapse numbers compared to the untreated control (*p* = 0.9555; Fig. 4). Notably, co-treatment with 1 μM N-Aβcore rescued the synaptic loss caused by 1 μM Aβ_1–42_ alone to levels similar to that of the untreated control (*p* = 0.9999; Fig. 4), suggesting the N-Aβcore may be able to protect synapses from oligomeric Aβ-induced synaptotoxicity.

**Fig. 5:**
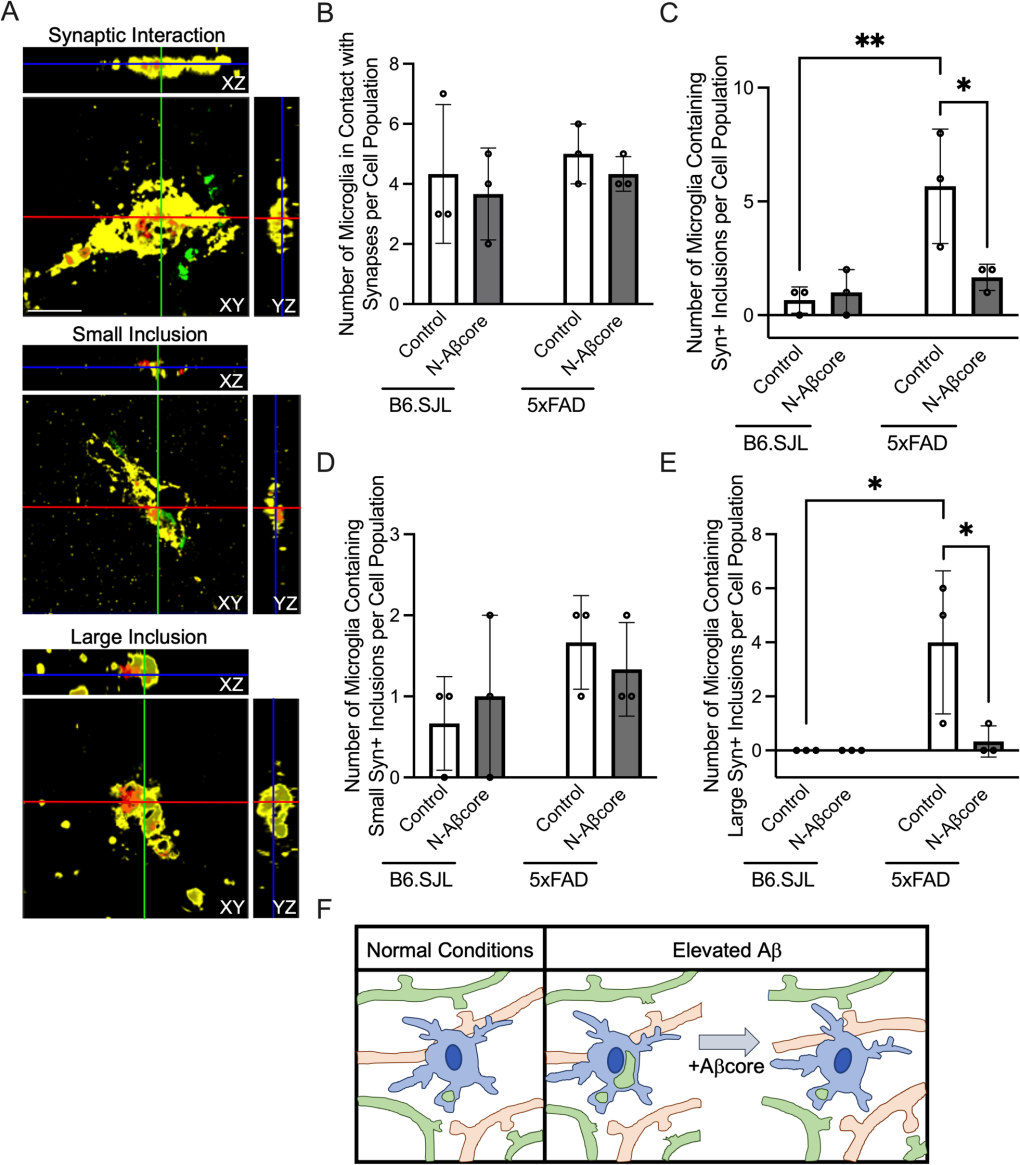
N-Aβcore protects against engulfment of presynaptic elements in 5xFAD mice.** A** Representative orthogonal views of Iba1 (yellow) and CD68 (red) labeled microglia in contact with or containing synaptophysin-positive (Syn +) inclusions (green) in 3-month-old B6SJL or 5xFAD organotypic slice cultures counterstained with DAPI after 7 days daily treatment with media only (Control) or 1 μM N-Aβcore. Images obtained on a Leica SP8 confocal microscope using a 63X objective. Scale bar: 10 μm. **B** The number of Iba1 + CD68 + labeled microglia in contact with Syn + presynaptic elements after treatment as described in A. (*n* = 150 per treatment group).** C** The number of Iba1 + CD68 + labeled microglia containing Syn + inclusions (*n* = 150 per treatment group). **D** The number of Iba1 + CD68 + labeled microglia containing small Syn + inclusions (*n* = 150 per treatment group). **E** The number of Iba1 + CD68 + labeled microglia containing large Syn + inclusions (*n* = 150 per treatment group). **F** Diagram depicting microglia-dependent synaptic pruning under normal (left panel) and elevated Aβ (right panel) conditions. The addition of 1 μM N-Aβcore attenuates the increase in synaptic pruning induced by elevated levels of Aβ. *n* represents the number of cells. Data points are averages from each experiment (*n* = 50 cells per experiment), with a total of *n* = 150 cells examined across 3 slices per treatment group in 3 separate experiments. Data are means ± SD, analyzed via one-way ANOVA with Dunnett post hoc test

To determine whether microglia-dependent synaptic engulfment is increased in 5xFAD mice and whether N-Aβcore treatment could alter microglial synaptic interaction or engulfment, the proportion of microglia containing synaptic elements from slice cultures taken from 3-month-old 5xFAD mice was compared to those from matched 3-month-old B6SJL slice cultures after 7 days of daily treatment with media only (Control) or 1 μM N-Aβcore (Fig. 5). The presynaptic vesicle-associated protein synaptophysin was utilized to tag presynaptic elements (green).

Microglia were double labeled with Iba1 (yellow) and CD68 (red), as the dual expression of these increases in activated phagocytic microglia [38, 39]. The microglia from each group were then scored into three separate categories: (1) no synaptic interaction, (2) synaptic interaction, (3) containing synaptophysin-positive (Syn +) inclusions (see “Materials and methods”). The population of microglia interacting with, but not engulfing, Syn + elements remained similar between the 5xFAD and B6SJL slices and was not altered by N-Aβcore treatment (Fig. 5B). However, interestingly, there was a significant increase in population of microglia containing Syn + inclusions in the control 5xFAD slices compared to the control B6SJL slices (Fig. 5C), which was attenuated by 1 μM N-Aβcore treatment.

To examine the population of Syn + containing microglia in more detail, the microglia containing Syn + inclusions were further scored in two different categories based on the size of the Syn + inclusions (see “Materials and methods”). Scoring the population of Syn + inclusion containing microglia in this manner revealed that the population of microglia containing small Syn + inclusions remained similar between the 5xFAD and B6SJL strains treated with or without N-Aβcore (Fig. 5D). Interestingly, the average diameter of the small Syn + synaptic inclusions (1.44 μm ± 0.37) and the Syn + synaptic puncta scored within the synaptic interaction group (1.65 μm ± 0.50) are not significantly different (*p* = 0.1417), suggesting these small Syn + synaptic inclusions may be individual Syn + puncta. By contrast, a dramatic increase in the population of microglia containing large Syn + inclusions (> 1 SD over the average Syn + inclusion size) in the 5xFAD slice cultures was observed compared to the B6SJL slices (Control groups; Fig. 5E), with substantial colocalization with the CD68 (evident as brown staining). This enhanced microglial synaptic engulfment was mitigated by 1 μM N-Aβcore treatment (Fig. 5E, F). In a preliminary experiment, N-Aβcore treatment also significantly reduced the observed increase in overall C3 protein expression in 5xFAD organotypic slices compared to the genetic background B6.SJL (Additional file 1: Fig. S8). Altogether, these results suggest exogenous application of the N-Aβcore attenuates Aβ-induced synaptotoxicity and loss, possibly involving a reduction in the expression of the complement protein C3 and mitigating ongoing Aβ-induced synaptic loss by direct microglial engulfment.

### N-Aβ fragment and N-Aβcore protect against gliotoxicity induced by high levels of Aβ

To mimic the cytotoxicity and cellular changes in astrocytes and microglia induced by high pathogenic levels of Aβ, while residing within a proinflammatory environment leading to cytotoxicity, an in vitro model was developed in which primary cortical astrocytes and microglia in mixed glial cultures were exogenously treated with Aβ_1–42_ over a prolonged period (Additional file 1: Fig. S9). Populations of astrocytes can become functionally impaired in AD, with deficits in gene or protein expression [24, 65], and treatment of cultured astrocytes with the toxic C-terminal Aβ sequence, Aβ_25–35_, induced intracellular peroxide formation and decreased cell viability in a concentration-dependent manner [66]. In addition, Aβ_1–42_-treated microglia release fragmented and dysfunctional mitochondria into the neuronal milieu, activating nearby astrocytes into the A1 reactive state and triggering neuronal death [67, 68]. Oxidative stress, as the percentage of ROS-positive cells in the mixed glial population, was utilized to determine the concentration necessary to induce cytotoxicity in primary cortical astrocytes and microglia. The two highest concentrations tested, 2.5 μM and 5 μM Aβ_1–42_, induced a significant increase in the percentage of ROS-positive cells after 3 days of daily treatment compared to the untreated control (Additional file 1: Appendix, Fig. S9A). Furthermore, it was determined daily treatments with 2.5 μM Aβ_1–42_ for 2, 3, and 5 days induced significant oxidative stress, mitochondrial dysfunction and apoptosis in primary cortical astrocytes and microglia, respectively (Additional file 1: Appendix, Fig. S9B, D).

**Fig. 6:**
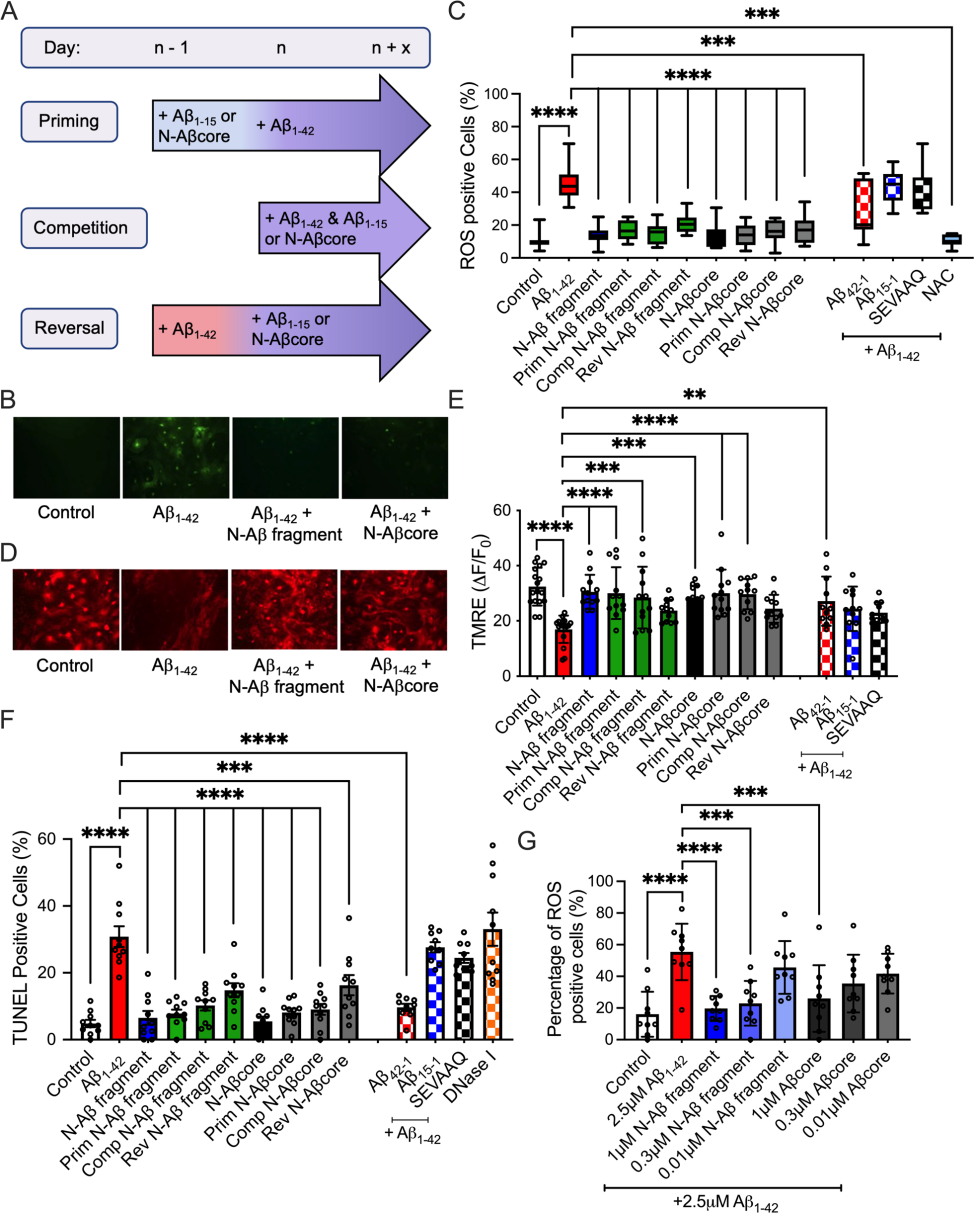
The N-Aβ fragment and N-Aβcore mitigate cellular toxicity induced by Aβ_1–42_ in primary cortical astrocytes and microglia. Primary cortical astrocytes and microglia in mixed glial cultures were treated daily with media only (Control), 2.5 μM Aβ_1–42_, 2.5 μM N-Aβ fragment, 2.5 μM N-Aβcore, 2.5 μM Aβ_1–42_ + 1 μM N-Aβ fragment or 2.5 μM Aβ_1–42_ + 1 μM N-Aβcore prior to analysis of cellular toxicity. **A** Treatment schematic depicting priming, competition and reversal conditions. N-Aβ fragment: Aβ_1-15_. **B** Representative images of reactive oxygen species (ROS) staining after daily treatment for two days with media only (Control), 2.5 μM Aβ_1–42_, 2.5 μM Aβ_1–42_ + 1 μM N-Aβ fragment or 2.5 μM Aβ_1–42_ + 1 μM N-Aβcore. **C** Percentage of ROS-positive glial cells under priming, competition and reversal conditions (*n* = 3 cultures). **D** Representative images of TMRE staining after daily treatment for three days with media only (Control), 2.5 μM Aβ_1–42_, 2.5 μM Aβ_1–42_ + 1 μM N-Aβ fragment or 2.5 μM Aβ_1–42_ + 1 μM N-Aβcore. **E** Mitochondrial membrane potential disruption was quantified by the integrated values (Δ*F*/*F*) for TMRE staining in individual glial cells after treatment under priming, competition and reversal conditions. **F** Percentage of TUNEL-positive astrocytes and microglia after treatment under priming, competition and reversal conditions. Quantification of oxidative stress, mitochondrial dysfunction and apoptosis as the percent of mean cell counts per experimental *n* (total number of independent experiments). **G** Percentage of ROS-positive glial cells after treatment with media only (Control), 2.5 μM Aβ_1–42_ and 2.5 μM Aβ_1–42_ + 1, 0.3 or 0.01 μM N-Aβ fragment or N-Aβcore (*n* = 3 cultures)*.* Data are represented as a box-and-whisker plots across 5–95 percentile range, with the lines indicating median values or means ± SD. (All data analyzed via one-way ANOVA with Dunnett post hoc test as compared to 2.5 μM Aβ_1–42_.) **p < 0.01; ***p < 0.001; ****p < 0.0001. All images in **B** and **D** were obtained on an Olympus IX71 fluorescent microscope via a 40× objective. 2.5 μM Aβ_42-1_, 2.5 μM Aβ_1–42_ + 1 μM Aβ_15-1_ and 2.5 μM Aβ_1–42_ + 1 μM SEVAAQ (inactive substituted N-Aβcore) as additional control conditions. *Prim* priming condition, *Comp* competition condition, *Rev* reversal condition, *NAC* *N*-acetylcysteine, *ns* non-significant

We previously reported the non-toxic N-Aβ fragment and N-Aβcore protect against Aβ_1–42_-induced cellular toxicity in neurons [35]. To investigate whether the N-Aβ fragment and N-Aβcore also protect against gliotoxicity induced by Aβ_1–42_, the amounts of oxidative stress, mitochondrial dysfunction, and cell survival in primary astrocytes and microglia in mixed glial cultures were analyzed after Aβ treatment under priming, direct competition, or reversal conditions (see treatment schematic Fig. 6A). As in Additional file 1: Fig. S9, treatment of primary cortical astrocytes and microglia with 2.5 μM Aβ_1–42_ induced oxidative stress, mitochondrial membrane potential disruption and apoptosis (Fig. 6B–F; Additional file 1: Appendix, Fig. S10A, B), whereas the reverse sequence Aβ_42-1_ did not. Co-treatment with 1 μM of the N-Aβ fragment or N-Aβcore abolished or attenuated, respectively, the calcium response evoked by 2.5 μM Aβ_1–42_ alone (Additional file 1: Appendix, Fig. S3D), suggesting 1 μM co-treatments with either of the N-Aβ fragments may be able to mitigate Aβ_1–42_-induced cytotoxicity in astrocytes and microglia, as well. Indeed, the addition of 1 μM N-Aβ fragment or N-Aβcore mitigated the oxidative stress, mitochondrial dysfunction and apoptosis induced by 2.5 μM Aβ_1–42_ alone under priming or direct competition conditions back down to control levels (Fig. 6B–F; Additional file 1: Appendix, Fig. S10A, B). Moreover, the co-treatment with either of the N-Aβ fragments attenuated Aβ_1–42_-induced oxidative stress and apoptosis under reversal conditions (Fig. 6C F; Additional file 1: Appendix, Fig. S10A). Furthermore, co-treatment with the N-Aβ fragment or N-Aβcore mitigated the loss of cell viability in primary microglia, with a similar trend in primary astrocytes, as determined by direct cell counts (Additional file 1: Appendix, Fig. S10C). Under these conditions there was an initial upswing in astrocyte cell counts (0–5 days), owing to cell replication, later offset (after 5–10 days) by a downturn in astrocyte cell survival, enhanced by 1 μM Aβ_1–42_ as compared to untreated control cultures. A similar pattern was observed for microglia cell counts, but the impact of Aβ_1–42_ was decidedly more pronounced, leading to substantial cell death (Additional file 1: Fig. S10C). Importantly, treatment with either N-Aβ fragment or N-Aβcore alone did not induce cytotoxic effects over control levels and the control peptides Aβ_15-1_ and the inactive substituted Aβcore sequence SEVAAQ did not mitigate the gliotoxicity induced by co-treatment with Aβ_1–42_ (Fig. 6C, E, F; Additional file 1: Appendix, Fig. S10A, B). Taken together, the results indicate a strong cytoprotective action and sequence specificity of the N-Aβ fragment and N-Aβcore against Aβ_1–42_-induced gliotoxicity in astrocytes and microglia.

To examine the limits of the protective effects of the N-Aβ fragment and N-Aβcore against full-length Aβ_1–42_-induced oxidative stress in primary cortical astrocytes and microglia, the cells were co-treated with 2.5 μM Aβ_1–42_ and a series of dilutions of either the N-Aβ fragment or the N-Aβcore (Fig. 6G). Co-treatment with the lowest concentration tested, 0.01 μM of either the N-Aβ fragments did not prevent the Aβ_1–42_-induced oxidative stress (Fig. 6G). Surprisingly, however, treatment with an 8.3-fold lower concentration of N-Aβ fragment, 0.3 μM, on a molar basis, compared to full-length Aβ_1–42_ at 2.5 μM was able to significantly mitigate the oxidative stress induced by full-length Aβ_1–42_ in morphologically identified primary astrocytes and microglia (Fig. 6G). The same trend was observed with co-treatment of 2.5 μM Aβ_1–42_ and 0.3 μM N-Aβcore, although the p-value did not reach significance (*p* = 0.05; Fig. 6G), suggesting the N-Aβ fragment is more potent than the N-Aβcore.

Finally, we sought to investigate potential mechanism(s) underlying the protective effects of the N-Aβ fragments. The major receptors regulated by Aβ on neurons include cellular prion (PrP^C^), nicotinic acetylcholine receptors (nAChRs) and NMDA-type glutamate receptors (reviewed in [69]). Under normal conditions, astrocytes and microglia express NMDA receptors, PrP^C^, as well as α7- and α4-subtypes of nAChRs [70–75]. As a preliminary experiment to determine the mechanism(s) through which the N-Aβ fragment and N-Aβcore exert their protective effects, we examined the capability of these N-Aβ fragments to mitigate the Aβ_1–42_-induced increase in oxidative stress in primary astrocytes and microglia utilizing selective antagonists for PrP^C^, α7-nAChaRs or α4-nAChRs (Additional file 1: Fig. S11). In agreement with other studies [76, 77], the inhibition of PrP^C^, α7-nAChRs or α4-nAChRs all significantly decreased the extent of Aβ_1–42_-induced oxidative stress in primary astrocytes and microglia (Additional file 1: Fig. S11). Inhibition of these receptors also partially reversed the protective functions of the N-Aβ fragment and the N-Aβcore (Additional file 1: Fig. S11, Table S1). Interestingly, the application of the anti-PrP^C^ antibody 6D11 significantly increased the amount of oxidative stress present in astrocytes and microglia in the Control group treated with media only compared to the Control group without any antibody or drug present (*p* = 0.0083; Additional file 1: Fig. S11), suggesting the PrP^C^ receptor and the pathways it regulates may be important to the reduction of oxidative stress within astrocytes and microglia. Collectively, these results suggest the N-Aβ fragment or N-Aβcore mitigate full-length Aβ-induced oxidative stress via interaction with PrP^C^ and α4- or 7-nAChRs.

## Discussion

Increasing evidence implicates microglia in playing an active role in synaptic loss in AD. Astrocytes have also been shown to phagocytose excitatory synapses in vivo in the hippocampus of adult mice using fluorescent phagocytosis reporters [78] and may contribute to synaptic loss in AD as well, though this remains to be fully investigated. Microglia express many immunological receptors capable of recognizing both pathogens and oligomeric or fibrillar Aβ (reviewed in [79]). The activation of these receptors upregulates the production of proinflammatory mediators, which have been shown to disrupt the formation of dendritic spines [80], induce synaptic loss [81, 82] and neuronal death indirectly through the activation of astrocytes into an A1 activation state [20]. In addition, reactive microglia have also been directly implicated in synaptic removal via engulfment during development [82, 83] and in AD [16]. C1q and C3, upregulated in human AD brain tissues and CSF [84, 85] as well as in aged AD transgenic mice [86, 87] may opsonize synapses in the presence of oligomeric Aβ for removal by microglia [16, 88], as exposure of microglia in adult mice to oligomeric Aβ induced a significant increase in microglia-mediated engulfment of synaptic material [16]. Moreover, APPPS1 mice lacking C3 had fewer plaque-associated astrocytes and microglia, improved cognitive function, and were protected from an age-dependent loss of synapses and neurons, despite an increase in Aβ plaques compared to APP/PS1 mice expressing C3 [89], suggesting the aberrant upregulation of the complement system can contribute to AD disease progression. Here, we demonstrated that exogenous application of N-Aβcore mitigates the upregulated hippocampal C3 expression in 3-month-old 5xFAD organotypic coronal slice cultures (Fig. S8), suggesting the N-Aβcore may be able to reduce complement-dependent synaptic loss in these mice. Indeed, we observed here a dramatic increase in the population of microglia containing Syn + inclusions in 3-month-old 5xFAD organotypic coronal brain slice cultures compared to aged-matched wild-type slice cultures, suggesting activated microglia may be important players for the known reduction in synaptophysin levels in 5xFAD mice at 4 months of age [37], consistent with loss of presynaptic terminals.

A more refined analysis revealed that this increase in the 5xFAD coronal brain slice cultures was primarily due to increased numbers of microglia containing Syn + inclusions substantially larger than the average presynaptic bouton, and which were primarily colocalized with the lysosomal protein CD68, possibly indicating accumulation in the lysosomal pathway. That this increase in large Syn + inclusions in the 5xFAD brain slice cultures was fully attenuated by treatment with the N-Aβcore has several possible implications. First, the attenuation of the increase by the N-Aβcore within a timeframe of days in the absence of any differences in synaptic contact with microglia or small inclusions indicates a substantial rate of ongoing synaptic engulfment. Second, the accumulation of large inclusions in the microglia in the 5xFAD brain slices indicates that the high levels of APP/Aβ in this model may have altered the rate of engulfment and/or the rate of digestion of the engulfed terminals in the lysosomal pathway. A substantial increase in the rate of engulfment in response to elevated Aβ is consistent with previous studies [16]. Third, that an increase in the rate of engulfment was not manifested as a significant increase in small Syn + inclusions in the 5xFAD slice cultures over that seen for wild-type slices (though there was a trend) may indicate that the engulfed terminals are rapidly directed to the lysosomal pathway, which then became overwhelmed in the 5xFAD microglia resulting in large inclusions. The latter is consistent with pathological accumulation of Aβ in this mouse model leading to expression of proinflammatory cytokines, such as IL-1β, shown to impair microglial clearance of Aβ and neuronal debris [90, 91]. Microglia express various cellular receptors that can recognize soluble oligomeric or aggregated Aβ including TLRs, NLRs, formyl peptide receptors, and scavenger receptors [92]. One such receptor, receptor for advanced glycation end-products (RAGE), is a type of scavenger receptor found on microglia [93] that is known to enhance Aβ-induced activation of these cells into a proinflammatory phenotype, mediate the induction of proinflammatory mediators including IL-1β and TNFα and increase microglial migration [93]. The association between Aβ and microglial RAGE also induces the phosphorylation of p38 MAPK and c-Jun N-terminal kinase (JNK), leading to the secretion of IL-1β and synaptic dysfunction [94], suggesting that RAGE-dependent signaling in microglia is involved in synaptic degeneration which may in turn promote synaptic phagocytosis by microglia. Here, we demonstrate exogenous application of the N-Aβcore mitigates the Aβ-induced phenotypic shift of microglia into an activated state (Fig. 2), reduces the expression and/or release of proinflammatory markers from BV2 microglial cells (Fig. 3), and attenuates neuronal synaptic loss (Fig. 4), indicating the N-Aβcore may interfere with the activation of RAGE or other microglial receptors that can recognize various forms of Aβ, though this remains to be determined. Future studies using live-cell imaging will be necessary to determine the specific mechanism(s) underlying these findings, including the extent to which attenuation of complement-linked opsonization plays a role.

The tissue concentration of Aβ impacts the structure of the peptide, and a rise in Aβ tissue concentration drives the oligomerization and aggregation propensity of Aβ [53]. The hydrophobic C-terminal domain of Aβ is largely responsible for the formation of low- and high-soluble oligomeric Aβ species through the step-wise self-association of anti-parallel beta sheets [8, 33], while the hydrophilic N-terminal domain (residues 1–14) of Aβ, from which the N-Aβ fragments are derived, remains largely unstructured [32]. One possible mechanism by which the N-Aβ fragments induce their neuroprotective effects might have been by binding to oligomeric Aβ directly, sterically hindering the action of toxic Aβ on the astrocytes and microglia. However, several lines of evidence argue against this mechanism. First, on a structural level, the N-Aβ fragments failed to alter Aβ aggregation/oligomerization and the N-Aβcore was found to dock into the α7-nAChR ligand-binding domain [35]. Second, N-Aβ fragments were shown to induce directly neuronal Ca^2+^ transients [35], as was also shown here for glia (Additional file 1: Fig. S3). Third, the N-Aβ fragments were found to be able to reverse Aβ-induced apoptosis well after neuronal cells committed to the cell death process as noted by DNA fragmentation [35]. Lastly, the peptides were able to reverse the prolonged impact of elevated Aβ to alter synaptic plasticity in 5xFAD mouse preparations from which Aβ was thoroughly washed out [36]. Lastly, the N-Aβ fragments were shown to prevent glutamate neurotoxicity [35], independent of Aβ. Future experiments will further investigate the molecular mechanism(s) by which the phenotypic and gliotoxic effects of Aβ on astrocytes and microglia are mitigated by the N-Aβ fragments (e.g., anti-apoptotic pathways; gene regulation; synaptic pruning).

The aforementioned neuroprotective and glioprotective actions of the N-Aβ fragments implicate cellular receptors for the peptides on astrocytes and microglia. The α7-nAChR subtype has been found to be particularly important in AD. An increase in the expression of α7-, but not α4-, nAChRs has been observed in cortical and hippocampal astrocytes from AD patients compared to age-matched controls [95], and Aβ can activate or inhibit astrocytic α7-nAChRs in a dose-dependent manner [75]. Aβ under physiological conditions [96–98] was found to enhance spontaneous astrocyte calcium transients and regulate neuron–glia signaling in an α7-nAChR-dependent manner, the dysfunction of which can contribute to glia-based aspects of AD pathogenesis [99]. Higher Aβ concentrations (into the μM range) increase astrocytic α7-nAChR expression and intracellular calcium release from intracellular stores, potentially contributing to a number of inflammatory cascades [92]. Furthermore, TNFα release and microglial activation are attenuated in a dose-dependent manner through an α7-nAChR-dependent mechanism [99], though prolonged Aβ interaction may block or chronically inactivate microglial α7-nAChRs, allowing for an aberrant release of TNFα and rampant neuroinflammation [100]. Direct and indirect evidence indicates that N-Aβ fragment and N-Aβcore bind to known receptors regulated by Aβ, such as PrP^C^, NMDA and nAChRs [34, 35, 69]. Here, we demonstrate that selective antagonism of α7-nAChR activity by 10 nM MLA [101] and α4-nAChR activity by 100 nM DHBE [102] compromised the protective functions of the N-Aβ fragment or N-Aβcore against full-length Aβ-induced cytotoxicity, as assessed via the ability of these N-Aβ fragments to mitigate oxidative stress induced by Aβ_1–42_ (Additional file 1: Fig. S11), suggesting the N-Aβ fragments act through nAChRs on glial cells as well. In addition, we discovered that the action of the N-Aβ fragment and N-Aβcore involves another known Aβ target receptor, PrP^C^. These results suggest the N-Aβ fragments interact with multiple known Aβ target receptors, though a more thorough investigation into the binding interactions between the N-Aβ fragment or N-Aβcore and these or other prominent receptors on astrocytes and microglia needs to be conducted in future studies to elucidate the true mechanism of action of these N-Aβ fragments.

One caveat to this study is the use of ex vivo organotypic slice cultures from transgenic 5xFAD mice, a model based on familial AD (FAD) mutations. The majority of AD mouse models utilize FAD mutations, though some non-familial AD mouse models have been generated as well. One such non-familial AD model, a wild-type human APP knockin mouse created by Serneels and colleagues, has an increased expression of Aβ [103]. Though this human APP knockin model, and others, lack other AD endophenotypes (e.g., Aβ plaques) [103], future studies should include a non-familial AD mouse model, as a separate control to determine the impact of familial AD mutations, as noted by the authors [103]. Nonetheless, for the present study focusing on glia, the ex vivo organotypic 5xFAD slice cultures offered a highly accessible, intact model system in which the basic structure and organization of the brain region of interest are retained, allowing for the study of the glial cells in the context of a complex cellular network. Moreover, the use of this model system allows for greater experimental control over the temporal application and concentration of the N-Aβ fragment or the N-Aβcore present in the media of our various experimental conditions. Although the uptake and clearance rates of full-length human Aβ have been measured in cerebrospinal fluid in vivo [3], the distribution and clearance have yet to be determined for the various fragments of Aβ, limiting control of the in vivo application of the N-Aβ fragments into the brain.

Activated astrocytes and microglia serve as a double-edged sword, playing many beneficial roles in the earlier stages of the disease and steadily converting to more detrimental roles with disease progression, eventually manifested as neuroinflammation. The continual accumulation of Aβ in AD pathogenesis induces significant increases in [Ca^2+^]_i_ in astrocytes and in microglia [10, 11], resulting in the secretion of proinflammatory cytokines, including IL-1β and TNFα, from these cells [21], as confirmed here for microglia using the model BV2 line (Fig. 3), in a feed-forward mechanism that skews their activation state to a proinflammatory phenotype that exacerbates neuronal death [104, 105]. Curiously, we also observed an increase in the secretion of the potentially protective neurotrophin BDNF. It is likely that this involves the same mechanism driving increased proinflammatory cytokine secretion at the concentration of Aβ used and timeframe studied, and that this represents an initial compensatory response, later overcome as Aβ continues to elevate and foster the neuroinflammatory response, eventually driving gliotoxicity. Importantly, we show that the N-Aβcore decreases the expression and release of several proinflammatory mediators, even under reversal conditions, in the presence of Aβ_1–42_ in BV2 microglial cells. In addition, we demonstrated that the non-toxic N-Aβ fragment and N-Aβcore were able to mitigate the activation of cortical astrocytes and microglia in vitro and the N-Aβcore attenuates the reactive gliosis observed in ex vivo aged 5xFAD organotypic slice cultures with substantial elevated levels of human Aβ. These findings are likely the result, largely, of the attenuation of the robust sustained intracellular calcium signals evoked by μM concentrations of Aβ_1–42_ by the N-Aβ fragment and N-Aβcore (Fig. S3 *D*). In addition, there is cross-talk between astrocytes and microglia via IL-3, which promotes microglia-based attenuation of Aβ pathology in AD [106], raising the possibility that the protective action of the N-terminal Aβ fragments may also involve downstream regulation of astrocyte–microglia interactions, which will be considered in future studies, as well.

The understanding of the physiological roles of Aβ, and especially fragments thereof, in neurons as well as glial cells remains limited. Aβ levels in the normal brain is estimated to be in the low picomolar range (~ 250 pM)  [97, 98]. Here, we demonstrate application of a physiologically relevant concentration of 100 pM of either Aβ, N-Aβ fragment or N-Aβcore elicits similar weak intracellular calcium responses within astrocytes and microglia and does not activate these cells, in noted contrast to that found for neurons and presynaptic terminals [34–36, 40, 41]. It may be that pM concentrations of these Aβ peptides, evoke small but similar modulatory effects through various biological pathways via the induction and coupling of modest intracellular calcium responses, though this would best be assessed through a thorough investigation into alterations in various biological pathways in astrocytes and microglia at the transcriptional and protein levels. In line with our findings, Lee et al. observed enhanced spontaneous oscillating astrocyte Ca^2+^ transients with the application of monomeric 200 pM Aβ_1–42_ [98], but no such modulatory effect with application of 200 pM oligomeric Aβ_1–42_, further suggesting a modulatory role for Aβ that we propose would be through the activity contained within residues 10–15, under normal conditions.

**Fig. 7:**
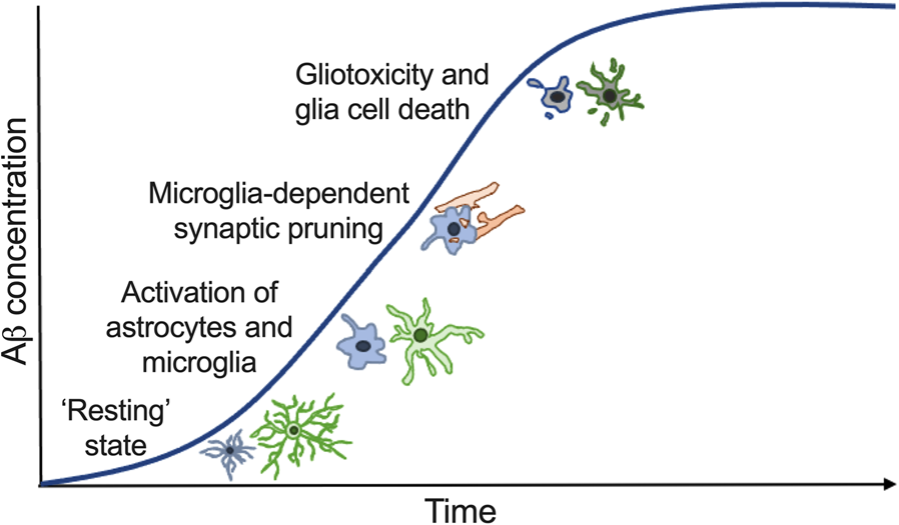
Astrocyte and microglia phenotypic state at increasing Aβ concentrations. Diagram depicting the change in astrocyte and microglia phenotypic state and function with increasing Aβ concentrations during AD. Astrocytes and microglia perform various neuromodulatory and neuroprotective functions in a surveillant, ‘resting’ state at low, physiological Aβ concentrations. Increasing Aβ concentrations activate astrocytes and microglia into reactive phenotypic states, inducing the production and release of various proinflammatory mediators and an increase in microglia-dependent synaptic pruning, exacerbating neuronal death. As the concentration of Aβ continues to rise during the course of AD, pathogenic Aβ levels induce cellular dysfunction, gliotoxicity, and cell death in astrocytes and microglia

The pathological role of Aβ on glial cells also remains to be fully investigated, though it has been hypothesized that the concentration of Aβ in AD likely reaches a threshold at which it becomes toxic to astrocytes and microglia, in addition to neurons, causing glial cell death [107, 108]. This gliotoxic intracellular Aβ concentration remains to be characterized. Here, we found exogenous treatment of 2.5 μM Aβ_1–42_ to be the lowest concentration necessary to induce significant cytotoxicity in cultured astrocytes and microglia (Additional file 1: Fig. S9A). The pathological accumulation of Aβ in AD also diminishes the capacity of astrocytes and microglia to perform their various important neurosupportive functions, either by converting these cells to various activated phenotypes or by inducing cytotoxicity, which contributes to disease progression (see Fig. 7). Our results further indicate the protective functions of the N-Aβ fragment and N-Aβcore against the cytotoxicity induced by μM concentrations of oligomeric Aβ_1–42_ extend beyond neurons [35] to astrocytes and microglia. Moreover, our demonstration that the N-Aβcore consistently attenuated or reversed Aβ-triggered phenotypic shifts to reactive microglia and astrocytes in the models under study provides a primary mechanism for protection by the N-Aβcore hexapeptide against the impact of reactive gliosis on synaptic changes with Aβ pathology.

All together, these results suggest the non-toxic N-Aβ fragment and N-Aβcore may provide an avenue for the development of novel therapeutics to hinder the development of chronic neuroinflammation in AD, limit the extent of Aβ-induced gliotoxicity to maintain the neurosupportive and neuroprotective functions inherent to astrocytes and microglia, and importantly, reduce microglia-dependent synaptic loss to slow the progression of AD.

## Conclusions

Persistent reactive astrogliosis and microgliosis leading to the development of chronic neuroinflammation are important contributors to disease progression in AD. Here, we show an endogenous N-terminal Aβ peptide (Aβ_1–15_: N-Aβ fragment) and an active core hexapeptide residing within this sequence (Aβ_10–15_: N-Aβcore) attenuate the Aβ-induced reactive astrogliosis and microgliosis in vitro in cultured mixed glial cultures and in ex vivo aged 5xFAD organotypic slice cultures, protect or reverse Aβ-induced gliotoxicity, reduce the expression and release of several proinflammatory mediators, and reverse enhanced microglia-mediated synaptic engulfment in ex vivo 5xFAD organotypic slice cultures. These findings may have important implications for the development of potential therapeutics that ablate the chronic neuroinflammation and microglia-mediated synaptic loss in AD to slow disease progression.

## Materials and methods

### Animals

All animal procedures (handling, use and euthanasia) were performed in accordance of an Institutional Animal Care and Use Committee-approved protocol (Ethical approval reference: 16-2282-4/5), compliant with National Institutes of Health (NIH) and Society for Neuroscience guidelines for the use of vertebrate animals in neuroscience research. The human APP/presenilin 1 (PSEN1) mutant transgenic mouse line, 5xFAD (Tg6799), on the B6SJLF1/J background (B6SJL-Tg(APPSwFlLOn,PSEN1*M146L*L286V) 6799Vas/Mmjax (from JAX stock #100012, MMRRC034840, Homozygous) was used as an extensively characterized model for Aβ-based pathology and neurodegeneration [37], along with age-matched genetic background (control wild-type) mice (B6SJL; MMRRC034840 Non-carrier). Mice were housed in ventilated cages with environmental enrichment in the John A. Burns School of Medicine (JABSOM) AAALAC-accredited Vivarium with ad libitum access to food and water. Roughly equal numbers of male and female mice were used and there were no apparent differences by sex. Mice were killed at the age of 0–2 days, 1 month, 2.5 months or 6.5 months. Inclusion/exclusion criteria were based on animal health.

### Organotypic slice cultures

Coronal slices containing cortices and hippocampi were prepared from 1-, 2.5- or 6.5-month-old human APP/PSEN1 mouse line, 5xFAD (Tg6799), or control (B6SJL background) mice, as described [109]. Following cervical dislocation and rapid decapitation, brains were isolated and subsequently sliced coronally into 200 μm sections using a vibratome (Leica VT 1200S SN# 10021) in ice-cold, sterile-filtered Slicing medium [Minimum Essential Medium (MEM) containing 13 mM NaHCO_3_, 25 mM HEPES, 5 mM Tris, 1.7 mM glucose and 3 mM MgCl_2_—pH 7.2]. Only slices containing fully intact dorsal hippocampi were selected for this study. Whole coronal slices containing cortex and hippocampi were plated onto membrane inserts (VWR catalog# 353090 or Millipore catalog #PICM0RG50) within 6-well plates containing 1.2 mL/well Feeding medium [Basal Medium Eagle (BME) containing 3% (v/v) Earle Balanced Salts Solution (EBSS), 20 mM NaCl, 5 mM NaHCO_3_, 0.2 mM CaCl_2_, 1.7 mM MgSO_4_, 48 mM glucose, 26.7 mM HEPES, 5% (v/v) heat-inactivated horse serum, 1% (v/v) penicillin/streptomycin, 0.255 mM ascorbic acid, 0.066% (v/v) human insulin—pH 7.2] and maintained in culture for 3 days in a 37 °C, 5% CO_2_, humidified environment prior to treatment. Media were changed after 48 h with fresh Feeding Medium. All treatments were performed in Feeding Medium without horse serum. Slice cultures were treated daily for 7 days with media only (untreated control) or 1 μM N-Aβcore.

### Murine cortical glia and neuron/glia culture

Cortical mixed glial cultures were prepared from neonatal BJSJL mouse pups (0–2 day old; one litter of 6–10 mice/preparation of either gender in roughly equivalent numbers) obtained from established colonies of wild-type B6SJL mice housed in the JABSOM AAALAC-accredited Vivarium, based on a modification of the protocol described in ref. 36 and similar to several other methods reviewed in ref. 110. Following rapid decapitation, brains were removed into sterile ice-cold HBSS for mixed glia cultures or Neurobasal A medium containing B-27 supplement, 5% fetal bovine serum and Gentamicin (Serum NB) for neuron/glia cultures. Cortices were then isolated under a stereomicroscope, minced and digested with papain in Hanks buffer with 10 mM cysteine at 37 °C for 15 min. The preparations were washed by centrifugation in sterile HBSS for mixed glia cultures or Serum NB for neuron/glia cultures. The isolated cell pellet was dissociated using sequential trituration with polished Pasteur pipettes of decreasing diameter and collected by low-speed centrifugation. The dissociated cells were resuspended and diluted to 1.9 × 10^5^ cells/mL.

To obtain a mixed glia-enriched cell population, the dissociated cells were plated in HBSS for 10–15 min at 37 °C in standard tissue culture dishes to separate out the non-adherent neuronal cells, which were removed by gentle washing. The glia-enriched adherent cells were then resuspended in 10% heat-inactivated horse serum in DMEM (Dulbecco’s modified Eagle media) containing 4.5 g/L glucose, without l-glutamine and with sodium pyruvate (Glia media) prior to being diluted to 1.9 × 10^5^ cells/mL in Glia media and plated into poly-d-lysine-coated 6-. 12-, or 24-well plates or coated glass coverslips (Horse serum is toxic to neurons). The glial cultures were maintained in Glia media for 7 days prior to the application of various treatments in a 37 °C, 5% CO_2_, humidified environment. Media were changed every 2–3 days with fresh Glia media. Analysis of the mixed glia cultures via immunostaining for glial (GFAP; Iba1/CD68) and neuron (NeuN) markers revealed a typical composition of 54% astrocytes, 37% microglia and 9% other cells (including neurons: < 2%).

For neuron/glia cultures, the dissociated cortical cells plated onto poly-d-lysine-coated glass coverslips were carefully washed once with Neurobasal A medium containing B-27 supplement and Gentamicin (Plain NB) prior to exchanging the culture medium for Plan NB supplemented with 10% glia conditioned media (CM) obtained from confluent mixed glia cultures. The cultures were maintained in Plain NB supplemented with 10% CM for 14 days prior to treatment in a 37 °C, 5% CO_2_, humidified environment. Media were exchanged every 2–3 days with fresh Plain NB supplemented with 10% CM.

CM recovered from mixed glia cultures was cooled on ice prior to centrifugation for 10 min at 1000 rpm at 4 °C to pellet any cells. Supernatants were removed and stored at -20 °C until needed.

### Microglial clonal cell culture

Murine BV2 microglial cells (RRID:CVCL_0182; courtesy of Dr. Jefferson Kinney, University of Nevada at Las Vegas) were used as a homogeneous model microglial cell system. The BV2 line was derived from mouse brain microglia that were retrovirally immortalized [55]. The cells were cultured in Dulbecco’s Modified Eagle Media (DMEM) with 10% fetal bovine serum (FBS) and 1% penicillin/streptomycin. Media were changed every 2–3 days and cells were maintained in a 37 °C, 5% CO_2_, humidified environment. BV2 microglial cells were immunostained according to the protocol for isolated cortical mixed glial cultures (see next section) using anti-rat CD68 (1:500; Abcam catalog # ab31630; RRID:AB_1141557), rabbit anti-TNFα (1:500; Abcam catalog # ab183218; RRID: AB_2889388), or rabbit monoclonal anti-iNOS (D6B6S) (1:200; Cell Signaling catalog # 13120; RRID:AB_2687529) primary antibodies.

### Immunocytochemistry

The quantification of the number of glial cells in a particular phenotypic state and neurons were determined via immunocytochemistry using fluorescently tagged antibodies to cell-type-specific markers in coronal slice cultures from B6SJL or 5xFAD mice or isolated cortical mixed glial cultures after Aβ treatments, as imaged using confocal microscopy. Coronal slice cultures isolated from 1-, 2.5- or 6.5-month-old mice (for glial markers), 10-month-old mice (for neuron markers) or isolated cortical mixed glial cultures (glial markers) were subjected to various treatments for 7 (slices) or 1–15 days (mixed glial cultures), respectively. Immunocytochemistry of slice cultures was performed according to the protocol described in [109]. In brief, cultures were fixed with freshly prepared 4% paraformaldehyde in phosphate-buffered saline (PBS; slice cultures: 5 min, primary cultures: 40 min). Slice cultures were subsequently incubated in 20% methanol in PBS for 5 min and washed once in PBS. Thereafter, cultures were permeabilized using Triton-X in Tris-buffered saline (TBS; slice culture: 0.05% Triton-X overnight at 4 °C, primary cultures: 0.1% Triton-X at room temp. for 30 min). Slices and adjacent surrounding membrane were carefully removed from the membrane inserts. Non-specific binding sites were blocked by incubation in a blocking buffer (slice culture: 20% bovine serum albumin (BSA) in PBS, primary culture: 5% BSA and 10% normal goat serum in TBS) overnight at 4 °C. Next, proteins of interest were labeled by an overnight incubation at 4 °C with primary antibodies in a blocking buffer containing 5% BSA (with the addition of 10% normal goat serum for primary cultures) in PBS. Primary antibodies utilized were anti-rat CD68 (1:200; Abcam), anti-mouse GFAP (1:200; Cell Signaling Technology catalog # 3670S; RRID:AB_561049), anti-rabbit Iba1 (1:200; Abcam catalog # ab178847; RRID:AB_2832244) and anti-NeuN (1:1,000; EMD Millipore, cat # ABN90; RRID:AB_11205592). The cultures were washed with 5% BSA in PBS for 30 min and incubated with the appropriate fluorophore-conjugated secondary antibody and DAPI to label cell nuclei for at room temperature (slice cultures: 4 h, primary cultures: 1 h), protected from light. Cultures were subsequently washed with PBS for 30 min, plated onto glass microscope slides, and sealed with Vectashield anti-fade mounting media (Vector Laboratories, catalog # H-1200; RRID: SCR_000821). The immunostained preparations were subsequently visualized using a Leica TCS SP8 confocal imaging system (for glia) using a 5×, 20×, 40× or 63× objective or a Leica Thunder wide-field microscope (for neurons).

### Enzyme-linked immunosorbant assay (ELISA) and dot-blot immunoassay

The concentration of the proinflammatory cytokine IL-1β and the neurotrophin BDNF released into the BV2 microglial cell culture media after various treatments were determined via ELISA using the Mouse IL-1β ELISA Kit (Abcam, catalog # ab229440) and a dot-blot immunoassay, respectively. In brief, BV2 microglial cells were subject to various treatments for 5–6 days. The medium for each condition was changed daily. At the end of each treatment period, the medium for each condition was recovered and immediately placed on ice prior to centrifugation for 10 min at 2000 rpm at 4 °C to pellet any cells. Supernatants were removed and stored at − 20 °C until needed. Protein concentrations in the supernatants were measured using the BCA assay.

For IL-1β, protein standards were prepared in double-distilled water to a final concentration of 0–200 pg/mL. 50 μL of sample or protein standard and 50 μL Antibody Cocktail were added to the appropriate wells of the microplate. The microplate was sealed and incubated for 1 h at RT on a plate shaker at 400 rpm. The wells of the microplate were subsequently washed three times in 350 μL/well 1× Wash Buffer PT. 100 μL/well CatchPoint HRP Developer solution was added to the appropriate wells and allowed to incubate for 10 min at RT on a plate shaker at 400 rpm, protected from light. Finally, the fluorescence readout was recorded on a SpectraMax M3 microplate reader at excitation/cutoff/emission of 530/570/590 nm using SoftMax Pro.

For BDNF, 8 μL of each supernatant was applied 1 μL at a time to a gridded nitrocellulose membrane and allowed to dry. A parallel set was produced as control. The membranes were incubated in 10% BSA and then processed for immunostaining using mouse anti-BDNF antibody (Abcam, cat #: ab205067; lot #GR3217060-14) at 1:10,000 final dilution in blocking buffer with 5% BSA, followed by washing with TBS-Triton. The membranes were then incubated with IRdye^®^ 680LT goat anti-mouse secondary IgG antibody (LI-COR, cat # 926-68020) in blocking buffer with 5% BSA and then extensively washed with TBS-Triton. The control membrane was incubated with secondary antibody only. The membranes were imaged on a LI-COR Odyssey IR imager. Immunostaining intensities were extracted from the scans using Image J. Intensity values for each sample were background (control)-subtracted and then normalized to total supernatant protein concentration.

### Reactive oxygen species (ROS)/Hoechst staining

Oxidative stress was determined via the production of reactive oxygen species (ROS) in individual cells using the Image iT LIVE Reactive Oxygen Species (ROS) Detection Kit (Invitrogen, catalog # I36007; RRID: SCR_008452) [111]. In brief, isolated cortical mixed glial cultures were subjected to various treatments for 1–3 days. The medium for each condition was changed daily. At the end of each treatment period, the cells were incubated with carboxy-H2DCFDA (component A) at 37 °C for 30 min. During the last 5 min of incubation, 2 μg/mL of Hoechst 33342 stain (component B) was added to assess, in parallel, the integrity of the cell nuclei. The cells were then washed with HBS. Finally, the cells were maintained in HBS and imaged live using an Olympus IX71 epifluorescence microscope at excitation/emission of 495/529 nm (ROS) and 350/461 nm (Hoechst 33342), respectively. Acquired images were analyzed via Image J (NIH, Bethesda, MD, USA), verifying the proportion of the fluorescent signals due to ROS via the use of N-acetylcysteine.

### Mitochondrial membrane potential

The mitochondrial membrane potential was measured using TMRE (tetramethylrhodamine ethyl ester)—mitochondrial membrane potential assay kit (Invitrogen, catalog # T669; RRID: SCR_008452) [35]. Briefly, isolated cortical mixed glial cultures were subjected to various treatments for 1–4 days. The medium for each condition was changed daily. At the end of the treatment periods, the cells were incubated with 50 nM TMRE and 2 μg/mL Hoechst 33342 stain in HBS for 20 min at 37 °C. The cells were subsequently washed with and maintained in HBS throughout live imaging using an Olympus IX71 epifluorescence microscope at excitation/emission of 549/575 nm (TMRE) and 350/461 nm (Hoechst 33342), respectively. Images were analyzed via Image J.

### Terminal deoxynucleotidyl transferase-mediated dUTP nick-end labeling (TUNEL) assay

Apoptosis was measured via TUNEL staining using the Click-iT^®^ TUNEL Alexa Fluor^®^ Imaging Assay (Invitrogen, catalog # C10245; RRID: SCR_00845) in accordance with the manufacturer’s protocol (see ref [111]). In brief, isolated cortical mixed glial cultures were subjected to various treatments for 3–7 days. The medium for each condition was changed daily. At the end of the treatment periods, the cells were fixed with freshly prepared 4% paraformaldehyde in PBS for 15 min and permeabilized with Triton X-100 (0.25% in PBS) for another 20 min. The cultures were then washed twice with de-ionized water and incubated with 50 μL of terminal deoxynucleotidyl transferase reaction buffer (Component A) for 10 min. The buffer was replaced with a TUNEL reaction mixture containing terminal deoxynucleotidyl transferase and incubated in a humidified chamber at 37 °C for 60 min. The cells were subsequently washed three times with 3% bovine serum albumin in PBS for 2 min per wash and thereafter, incubated with 50 μL of Click-iT reaction mixture (containing Alexa 488 azide) for 30 min at RT, protected from light. Afterwards, the cells were again washed with 3% bovine serum albumin in PBS and the cell nuclei were counterstained with 2 μg/mL Hoechst 33342 for 15 min at RT, protected from light exposure. The coverslips were finally washed twice with PBS before mounting onto a slide with Vectashield anti-fade mounting media and subsequently visualized using an Olympus IX71 epifluorescence microscope at excitation/emission of 495/519 nm (TUNEL) and 350/461 nm (Hoechst 33342), followed by analysis via Image J, verifying the proportion of apoptosis via the use of DNase I.

### Human Aβ and derivatives

Full-length human Aβ_1–42_, the N-Aβ fragment and Aβ_42-1_ were obtained from Bachem or AnaSpec as hydrochloride salts (Aβ_1–42_: catalog # 4045866.1; N-Aβ fragment: AS-61798; Aβ_42-1_: catalog # AS27076). The N-Aβcore (Aβ_10-15_: YEVHHQ) and the inactive substituted N-Aβcore (SEVAAQ) were custom-ordered from Peptide 2.0. All peptides were synthesized and isolated at > 98% purity, as assessed by mass spectrometry. Aβ (1–42), the N-Aβ fragment (1–15), and the N-Aβ core (10–15) and mutant forms were solubilized in double-distilled water and used at pM to μM final concentration in buffered saline as previously described [111]. All Aβ stock solutions were prepared as aliquots at 0.4 mM and stored at − 20 °C until use. Under these conditions the Aβ_1–42_ peptide is present as low-molecular-weight oligomers, whereas the N-Aβ fragment (Aβ_1-15_), and N-Aβcore are present in monomeric form. The reverse peptides Aβ_42-1_, Aβ_15-1_ and the inactive triple mutant (SEVAAQ) were utilized as additional controls, as noted in figure legends.

### Reagents

All standard reagents (buffers, salts, tissue culture media and substrates, paraformaldehyde, Triton X-100, etc.) were obtained from Sigma Aldrich, ThermoFisher or VWR and were of the highest grade available (> 98% purity). Heat-inactivated horse serum was purchased from Gibco (Catalog # 26080-088 Lot # 2180552). Tetrodotoxin was purchased from Abcam (Catalog # ab120054, Lot # GR169765). BD Cell-Tak was obtained from VWR (Catalog # 47743-684, Lot # 7100009). Papain was purchased from Worthington Biochemical (Catalog # LS003126 Lot # 30J20403). Poly-d-lysine was purchased from Sigma Aldrich (Catalog # A-003-E Lot # 90829-1).

### Data and statistical analyses

Digitized images obtained on the Leica TCS SP8 confocal microscope or Olympus IX71 fluorescent microscope were analyzed via ImageJ. Integrated fluorescence intensity values were extracted from individual cells from 3 randomly chosen fields of view, setting the black level to the same level obtained in an image of a culture incubated with secondary antibody alone for each paired condition using a primary and secondary antibody. Presynaptic boutons were identified by the localization of immunostaining for the presynaptic marker Bassoon apposed to postsynaptic dendrites visualized by immunostaining for Drebrin in merged images. All 0.5–2 μm puncta associated with primary, secondary or tertiary branches of dendrites in the plane of focus, clearly emanating from neuronal somata were counted per measured length. C3 expression was calculated using mean fluorescence intensity values obtained from the total hippocampal area from three individual organotypic slice cultures (2 hippocampi per slice) prepared from three separate mice. Microglia from each treatment group from 3-month-old 5xFAD or B6SJL (wild-type) were scored into three separate categories: (1) no synaptic interaction, (2) synaptic interaction, (3) containing synaptophysin-positive (Syn+) inclusions using the Leica LAS-X 3D Image Analysis software to visualize 3D projected images from compressed z-stacks. Co-labeling of Iba1 and CD68 (denoted as Iba1 + CD68 + microglia) using antibodies (rabbit and rat monoclonals, respectively) was used to label activated, phagocytic microglia [38, 39] and an antibody (mouse monoclonal) for the commonly used marker, synaptophysin was used to label presynaptic elements.

Microglia were scored based on the vicinity of and colocalization with Syn + presynaptic elements. Iba1 + CD68 + microglia with any number of Syn + presynaptic elements > 2 μm away were categorized as no synaptic interaction, any number of Syn + presynaptic elements ≤ 2 μm, but not colocalized with a Iba1 + CD68 + microglia were scored as “synaptic interaction”, while colocalization of any number of Syn + presynaptic elements and a Iba1 + CD68 + microglia were categorized as one microglia containing “Syn + inclusions”. Microglia containing Syn + inclusions were further separated into two categories based on the size of the Syn + inclusion: (1) small inclusions and (2) large inclusions. Large Syn + inclusions were defined as any single Syn + inclusion with a diameter greater than one standard deviation (SD) above the mean Syn + inclusion diameter. The values for the number of cells scored in each category were averaged for each of three experiments.

Treatment and units were randomized as to order for all assays and experiments. Biological replicates were based on independent samples (*n*). All experiments were repeated at least three times. For cell/soma area, process number, TMRE staining, and immunostaining fluorescence intensity experiments, image analysis was automated to reduce bias. For ROS staining and synaptic density experiments, image analysis was confirmed by a blinded observer. Quantitative results are presented as boxplots (5–95% confidence intervals), where appropriate, or means ± SD with overlaid raw data points. Statistical analyses were performed using Prism (GraphPad v5.0b; RRID:SCR_002798). Multiple comparisons were made using one-way ANOVA with Dunnett post hoc tests, or two-way ANOVA using Tukey post hoc tests, unless otherwise indicated. Paired comparison was made using Student’s *t*-tests. The minimum criterion for assessing changes in signals will be values that fall outside of the nominal variance of any background or baseline. *p*-values < 0.05 were considered the minimum for significance (as rejection of the null hypothesis).

## Supplementary Information

**Additional file 1**: **Extended Materials and Methods**. **Table S1**: Adjusted P values from oxidative stress in the presence of selective antagonists of PrPc, α7-nAChR or α4-nAChR pathways. **Fig. S1. **Treatment with 1 μM Aβcore reduces Aβ_1-42_-induced GFAP upregulation in astrocytes but not Iba1/CD68 upregulation in microglia from 7-month-old 5XFAD mice. **Fig. S2.** Treatment with 1 μM Aβcore increases neuronal populations in organotypic slice cultures from aged 5XFAD mice. **Fig. S3**: The N-Aβ fragment and N-Aβcore attenuate the Aβ_1-42_ calcium response in primary cortical astrocytes and microglia. **Fig.S4**. Treatment with 100 pM Aβ peptides does not induce an upregulation of GFAP expression in astrocytes or Iba1 andCD68 expression in microglia. **Fig. S5**. The N-Aβ fragment and N-Aβcore mitigate the upregulation of GFAP expression in astrocytes and Iba1/CD68 expression in microglia induced by 1 μM Aβ_1-42_. **Fig.S6**. N-Aβcore co-treatment reverses Aβ_1-42_-induced secretion of the neurotrophin BDNF in BV2 cells. **Fig. S7**. N-Aβcore co-treatment attenuates Aβ_1-42_-induced morphological changes and increased expression of CD68 in BV2 cells. **Fig. S8**. N-Aβcore mitigates complement C3 expression in organotypic slice cultures from 5xFAD mice. **Fig. S9**. Treatment with Aβ_1-42_ induces oxidative stress, mitochondrial dysfunction and apoptosis in primary cortical astrocytes and microglia in a dose- and time dependent manner. **Fig. S10**. N-Aβ fragment and N-Aβcore mitigate Aβ_1-42_-induced oxidative stress, mitochondrial dysfunction and cellular death in primary cortical astrocytes and microglia. **Fig. S11**. Selective antagonists of PrPc, α7-nAChR or α4-nAChR pathways partially compromise the protective functions of the N-Aβ fragment or N-Aβcore against the oxidative stress induced by full-length Aβ_1-42_ in primary cortical astrocytes and microglia.

## Data Availability

Summary data are available from the authors on request.

## Abbreviations

Aβ: Amyloid beta
AD: Alzheimer’s disease
APP: Amyloid precursor protein
BDNF: Brain-derived neurotrophin factor
BME: Basal Medium Eagle
BSA: Bovine serum albumin
C1q: Complement protein C1q
C3: Complement protein C3
Ca^2+^: Calcium
[Ca^2+^]_i_: Intracellular calcium concentrations
CD68: Cluster of differentiation 68
CM: Glia conditioned media
DMEM: Dulbecco’s modified Eagle media
EBSS: Earle balanced salts solution
ELISA: Enzyme-linked immunosorbent assay
F: Fluorescence intensity
F_0_: Fluorescence intensity at time 0
FBS: Fetal bovine serum
GFAP: Glial fibrillary acidic protein
HBSS: Hank’s balanced salt solution
Iba1: Ionized calcium binding adaptor molecule 1
IL-1β: Interleukin 1 beta
iNOS: Nitric oxide synthase
JABSOM: John A. Burns School of Medicine
LPS: Lipopolysaccharide
LTP: Long-term potentiation
MEM: Minimum essential medium
μM: Micromolar
nAChRs: Nicotinic acetylcholine receptors
NB: Neurobasal A medium
nM: Nanomolar
NO: Nitric oxide
PBS: Phosphate-buffered saline
pM: Picomolar
PrP^C^: Cellular prion protein
PSEN1: Presenilin 1
RAGE: Receptor for advanced glycation end-products
ROS: Reactive oxygen species
SD: Standard deviation
Syn+: Synaptophysin positive
TGFβ: Transforming growth factor beta
TMRE: Tetramethylrhodamine ethyl ester
TNFα: Tumor necrosis factor alpha
TUNEL: Terminal deoxynucleotidyl transferase dUTP nick-end labeling
