# Supplementary material for: The neuroprotective N-terminal amyloid-β core hexapeptide reverses reactive gliosis and gliotoxicity in Alzheimer’s disease pathology models
Source: J Neuroinflammation. 2023 May 27;20:129. doi: 10.1186/s12974-023-02807-9 (PMC10225108; doi:10.1186/s12974-023-02807-9)
Supplement: Supplementary file 1 — Additional file 1: Extended Materials and Methods. Table S1: Adjusted P values from oxidative stress in the presence of selective antagonists of PrPc, α7-nAChR or α4-nAChR pathways. Fig. S1. Treatment with 1 μM Aβcore reduces Aβ1-42-induced GFAP upregulation in astrocytes but not Iba1/CD68 upregulation in microglia from 7-month-old 5XFAD mice. Fig. S2. Treatment with 1 μM Aβcore increases neuronal populations in organotypic slice cultures from aged 5XFAD mice. Fig. S3: The N-Aβ fragment and N-Aβcore attenuate the Aβ1-42 calcium response in primary cortical astrocytes and microglia. Fig.S4. Treatment with 100 pM Aβ peptides does not induce an upregulation of GFAP expression in astrocytes or Iba1 andCD68 expression in microglia. Fig. S5. The N-Aβ fragment and N-Aβcore mitigate the upregulation of GFAP expression in astrocytes and Iba1/CD68 expression in microglia induced by 1 μM Aβ1-42. Fig.S6. N-Aβcore co-treatment reverses Aβ1-42-induced secretion of the neurotrophin BDNF in BV2 cells. Fig. S7. N-Aβcore co-treatment attenuates Aβ1-42-induced morphological changes and increased expression of CD68 in BV2 cells. Fig. S8. N-Aβcore mitigates complement C3 expression in organotypic slice cultures from 5xFAD mice. Fig. S9. Treatment with Aβ1-42 induces oxidative stress, mitochondrial dysfunction and apoptosis in primary cortical astrocytes and microglia in a dose- and time dependent manner. Fig. S10. N-Aβ fragment and N-Aβcore mitigate Aβ1-42-induced oxidative stress, mitochondrial dysfunction and cellular death in primary cortical astrocytes and microglia. Fig. S11. Selective antagonists of PrPc, α7-nAChR or α4-nAChR pathways partially compromise the protective functions of the N-Aβ fragment or N-Aβcore against the oxidative stress induced by full-length Aβ1-42 in primary cortical astrocytes and microglia. [file 12974_2023_2807_MOESM1_ESM.pdf]

Supplementary Information:

Extended Materials and Methods:

Live-Cell Calcium Imaging:

Changes in intracellular calcium ($[Ca^{2+}]_i$) levels in individual glial cells (morphologically identified astrocytes or microglia) from cortical mixed glia cultures were monitored by the Ca^{2+}-selective fluorescent dye Fluo-4/AM (Invitrogen, Carlsbad, CA, USA, catalog # F23917; RRID: SCR_008452). Mixed glia cultured were isolated and cultured on Cell-Tak-coated glass coverslips for 7-9 days until confluent, as described above. Fluo-4/AM was loaded into the mixed-glia cells and the cultures were perfused with oxygenated HEPES-buffered saline [HBS: 142 mM NaCl, 2.4 mM KCl, 1.2 mM K_2PO_4, 1 mM $MgCl_2$, 1 mM $CaCl_2$, 5 mM D-glucose, 10 mM HEPES (pH 7.4), 100 nM tetrodotoxin to suppress Na^+ currents] via a rapid exchange Warner perfusion chamber (Warner Instruments Model VC-6). Changes in fluorescent intensity (F) in response to various Aβ treatments were visualized by a Nikon PCM 2000 Chameleon confocal imaging system or a Leica TCS SP8 confocal imaging system via the 20X or 40X objective (see figure legends). Each time-series was normalized to baseline fluorescence intensity at time zero (F_0) to yield the relative change in $[Ca^{2+}]_i$ (as F/F_0). Peak responses for 4-10 cells per sample were collected during 540-580s after the initiation of stimulation and determined across all frames using Image J software.

Cell Survival:

Cell survival was determined by direct cell counts of GFAP-expressing astrocytes and Iba1-expressing microglia every day for 1-15 days of treatment. Glial cells were fixed with 4% paraformaldehyde, and immunocytochemistry was performed as noted above. For each treatment condition, all cells in 3 random fields of view were counted and averaged. The percent cell survival was calculated based on daily cell counts as a proportion of starting cell count numbers (i.e., Day 0): 100 x (average cells remaining each day / initial average cell number of

untreated control cells on Day 0). Only cells co-expressing DAPI-stained nuclei and GFAP or Iba1 were counted.

Supplementary Tables:

Table S1: Adjusted P values from Oxidative Stress in the Presence of Selective antagonists of PrP^c, α7-nAChR or α4-nAChR pathways

P Values		
Drug	Treatment	P value
No Drug	$A\beta_{1\text{-}42}$ vs. Control	<0.0001
	$A\beta_{1\text{-}42}$ vs. N-Aβ fragment	<0.0001
	$A\beta_{1\text{-}42}$ vs. N-Aβcore	<0.0001
	$A\beta_{1\text{-}42}$ vs. N-$A\beta_{1\text{-}42}$ + N-Aβcore	<0.0001
	$A\beta_{1\text{-}42}$ vs. N-$A\beta_{1\text{-}42}$ + N-Aβcore	<0.0001
6D11	$A\beta_{1\text{-}42}$ vs. Control	0.1153
	$A\beta_{1\text{-}42}$ vs. N-Aβ fragment	0.6072
	$A\beta_{1\text{-}42}$ vs. N-Aβcore	0.6335
	$A\beta_{1\text{-}42}$ vs. N-$A\beta_{1\text{-}42}$ + N-Aβcore	0.9918
	$A\beta_{1\text{-}42}$ vs. N-$A\beta_{1\text{-}42}$ + N-Aβcore	0.4292
MLA	$A\beta_{1\text{-}42}$ vs. Control	0.0317
	$A\beta_{1\text{-}42}$ vs. N-Aβ fragment	0.0506
	$A\beta_{1\text{-}42}$ vs. N-Aβcore	0.0924
	$A\beta_{1\text{-}42}$ vs. N-$A\beta_{1\text{-}42}$ + N-Aβcore	0.1135
	$A\beta_{1\text{-}42}$ vs. N-$A\beta_{1\text{-}42}$ + N-Aβcore	0.3476
DHBE	$A\beta_{1\text{-}42}$ vs. Control	0.4764
	$A\beta_{1\text{-}42}$ vs. N-Aβ fragment	0.9983
	$A\beta_{1\text{-}42}$ vs. N-Aβcore	0.3782
	$A\beta_{1\text{-}42}$ vs. N-$A\beta_{1\text{-}42}$ + N-Aβcore	0.9999
	$A\beta_{1\text{-}42}$ vs. N-$A\beta_{1\text{-}42}$ + N-Aβcore	0.1979

Supplementary Figures:

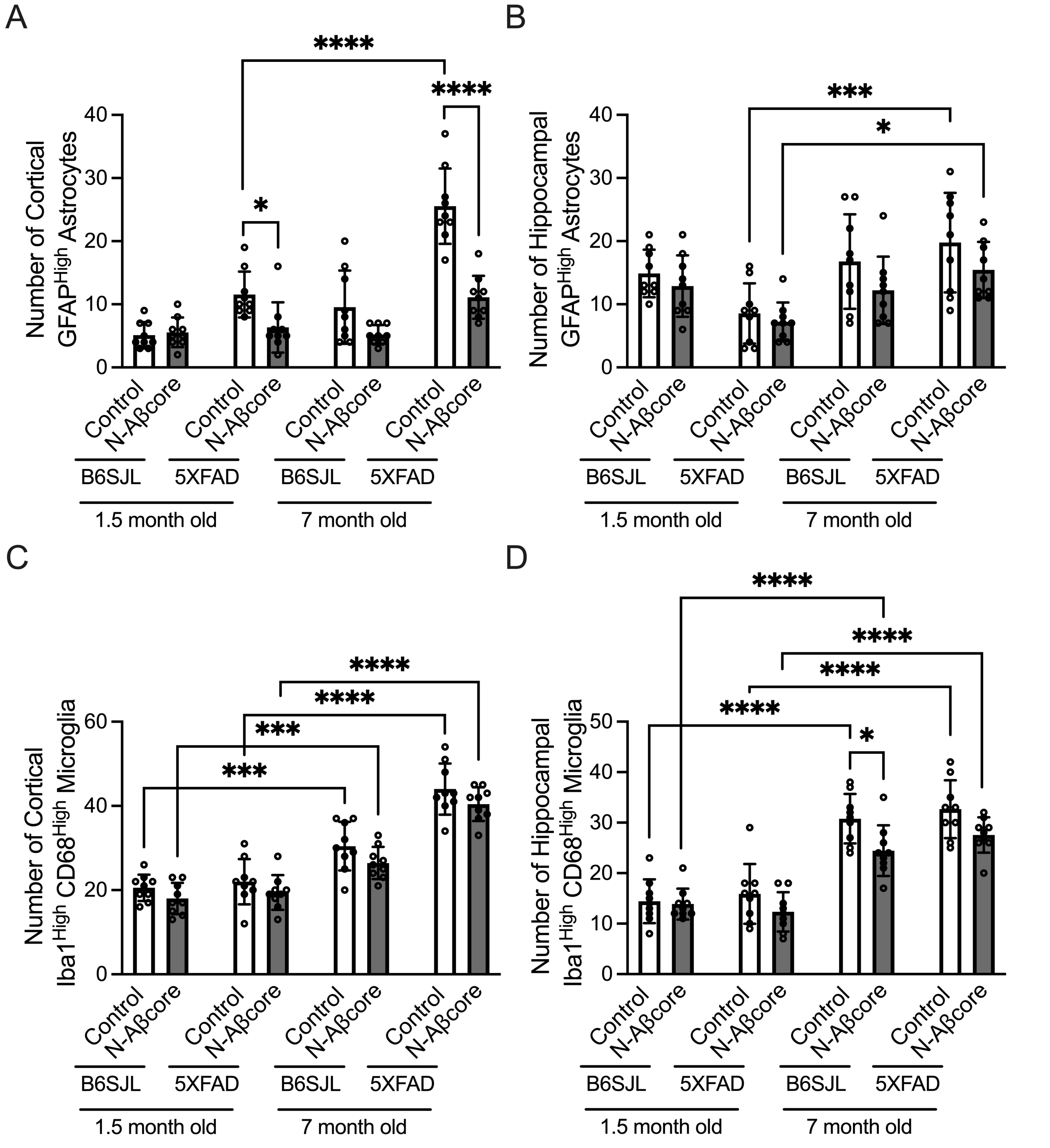

Fig. S1: Treatment with 1 μM Aβcore reduces $A\beta_{1\text{-}42}$-induced GFAP upregulation in astrocytes but not Iba1/CD68 upregulation in microglia from 7-month-old 5XFAD mice

Organotypic slice cultures from 1.5- and 7-month-old B6SJL (background) and 5XFAD mice were treated daily for 7 days with media only (Control) or 1 μM N-Aβcore. The number of cortical (**A**) and hippocampal (**B**) $GFAP^{High}$ expressing astrocytes. The number of cortical (**C**) and hippocampal (**D**) $Iba1^{High}$ and $CD68^{High}$ expressing microglia (*n*=100 cells across 6 slices from 3 mice per condition). Threshold for $Iba1^{high}$ and $CD68^{high}$: 1 SD over mean intensity value. All data analyzed via two-way ANOVA with Tukey *post hoc* test as compared to Control for each treatment day. *p<0.05 *** p<0.001 **** p<0.0001.

A

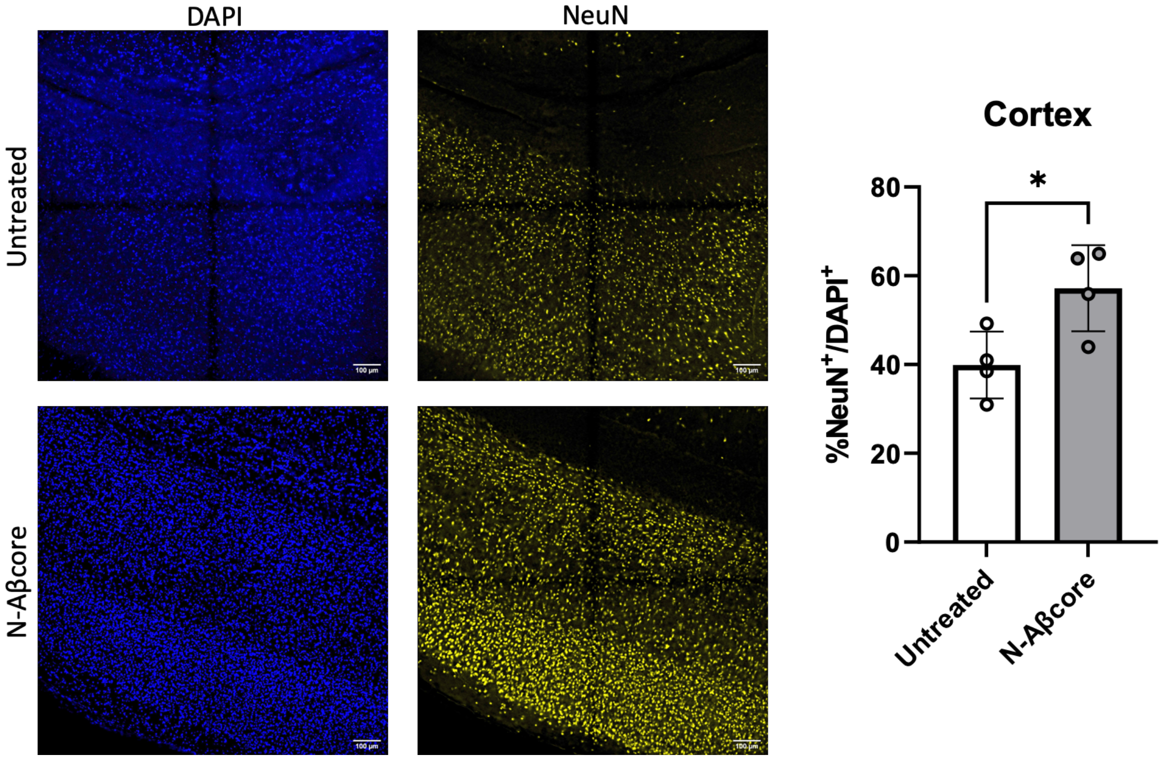

DAPI
NeuN
Untreated
N-Aβcore
Cortex
*
%NeuN+/DAPI+
80
60
40
20
0
Untreated
N-Aβcore

B

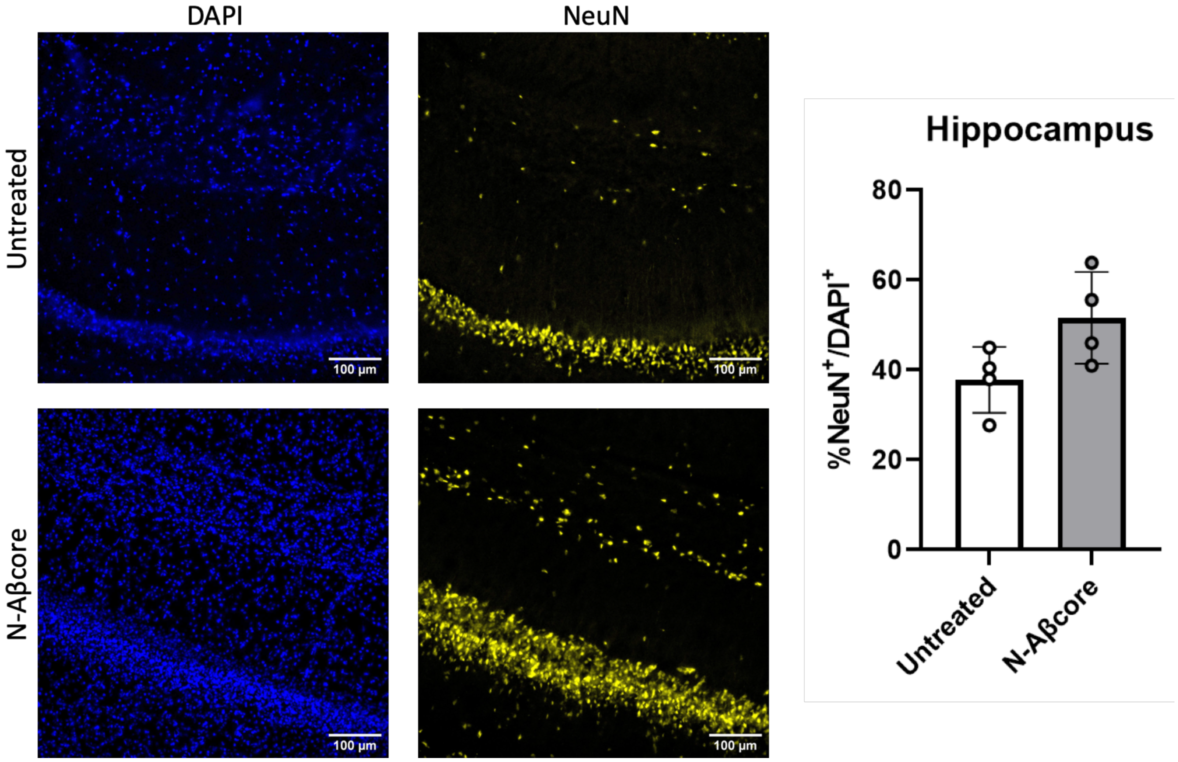

DAPI
NeuN
Untreated
N-Aβcore
100 μm
Hippocampus
%NeuN+/DAPI+
80
60
40
20
0
Untreated
N-Aβcore

Fig. S2: Treatment with 1 μM Aβcore increases neuronal populations in organotypic slice cultures from aged 5XFAD mice

Organotypic slice cultures from 10-month-old 5XFAD mice treated as described in the legend to Figure S1. The number of neurons were assessed using immunostaining for the neuronal marker NeuN in cortex (**A**) and hippocampus (**B**) imaged with a Leica Thunder microscope. Four adjacent ROIs were assembled from tile scanning for region within the brain slice. Data are expressed as % $NeuN^+$ cells out of the total cell population ($DAPI^+$), with individual points representing data from composite ROIs from separate culture slices (n=4). *p=0.031 two tailed, unpaired *t*-test; comparison for hippocampus (CA1) was not significant.

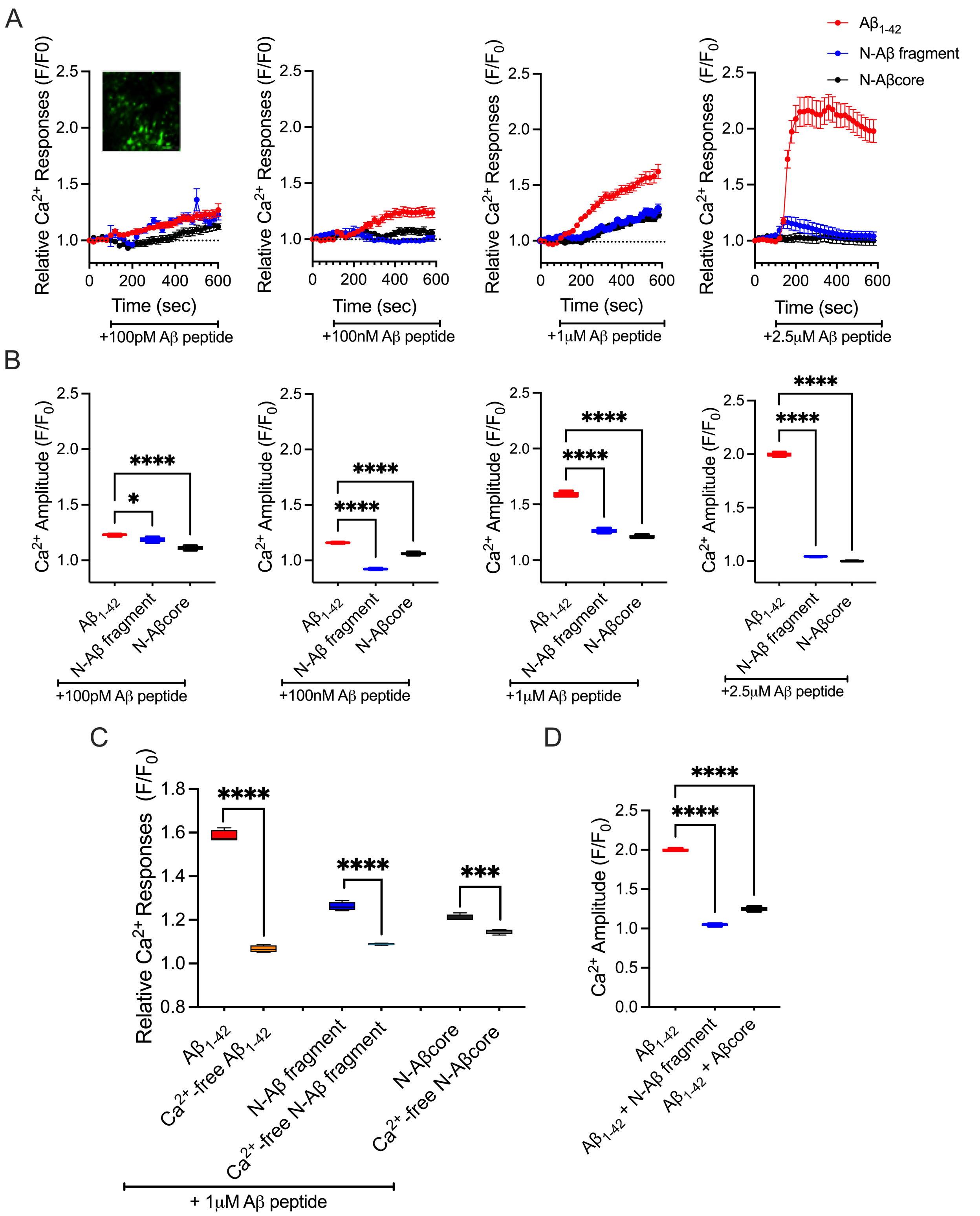

Fig. S3: The N-Aβ fragment and N-Aβcore attenuate the Aβ$_{1-42}$ calcium response in primary cortical astrocytes and microglia

A. Average normalized Ca^{2+} responses (F/F_0) in morphologically identified astrocytes and microglia from mixed glial cultures to 100 pM, 100 nM, 1 μM or 2.5 μM $A\beta_{1-42}$ (*n*=24-80), N-Aβ fragment (*n*=30-59) or N-Aβcore (*n*=28-52) for 10 minutes. Inset shows a representative image of a Fluo-4-loaded mixed glial culture. Amplitudes determined from averages of the last four timepoints (520-580 seconds) of normalized Ca^{2+} responses in A. (**B**), after 10-minute perfusion of 1 μM $A\beta_{1-42}$, N-Aβ fragment or N-Aβcore in HBS or Ca^{2+}-free HBS (**C**) and after 10-minute perfusion of 2.5 μM $A\beta_{1-42}$ (*n*=30), 2.5 μM $A\beta_{1-42}$ + 1 μM N-Aβ fragment (*n*=74) or 2.5 μM $A\beta_{1-42}$ + 1 μM N-Aβcore (*n*=60) (**D**). Averaged individual data points in A. are means ± SEM, F_0 = fluorescence at time 0, and *n* represents the total number of astrocytes and microglia examined. (Responses in roughly equivalent numbers of astrocytes and microglia were assessed.) Data in B-D are represented as a box-and-whisker plot across 5-95 percentile range with lines indicating median values. (C: Student's *t*-tests of averaged plateau values comparing each Aβ peptide in HBS to each Aβ peptide in Ca^{2+}-free HBS. B and D: Dunnett *post hoc* tests of averaged plateau values compared to $A\beta_{1-42}$). * p>0.05 *** p>0.001 **** p>0.0001

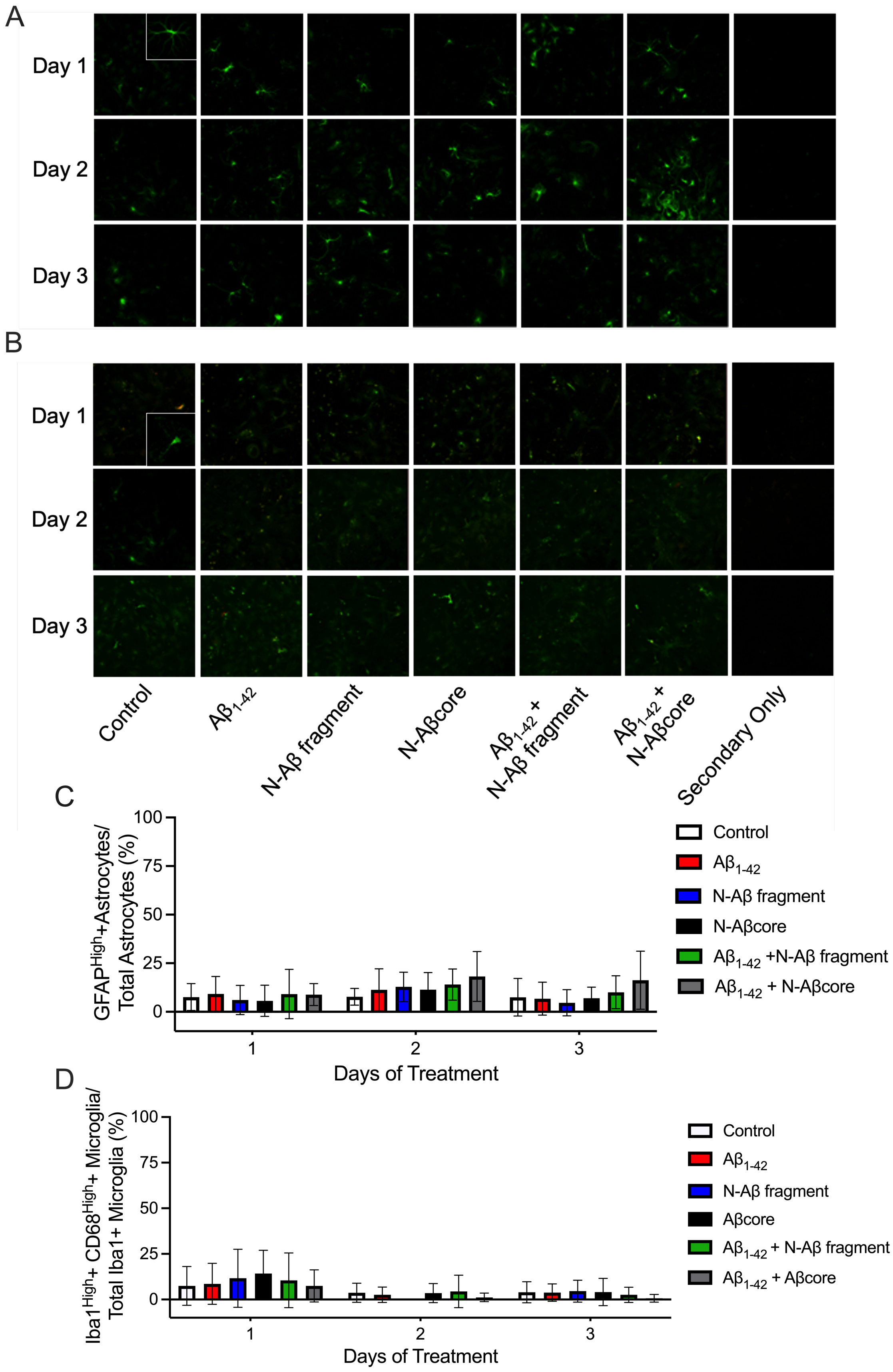

A
Day 1
Day 2
Day 3
B
Day 1
Day 2
Day 3
Control
Aβ1-42
N-Aβ fragment
N-Aβcore
Aβ1-42 +
N-Aβ fragment
Aβ1-42 +
N-Aβcore
Secondary Only
C
GFAPHigh+Astrocytes/
Total Astrocytes (%)
100
75
50
25
0
1
2
3
Days of Treatment
Control
Aβ1-42
N-Aβ fragment
N-Aβcore
Aβ1-42 +N-Aβ fragment
Aβ1-42 + N-Aβcore
D
Iba1High+ CD68High+ Microglia/
Total Iba1+ Microglia (%)
100
75
50
25
0
1
2
3
Days of Treatment
Control
Aβ1-42
N-Aβ fragment
Aβcore
Aβ1-42 + N-Aβ fragment
Aβ1-42 + Aβcore

Fig. S4: Treatment with 100 pM Aβ peptides does not induce an upregulation of GFAP expression in astrocytes or Iba1 and CD68 expression in microglia

Representative images of GFAP expression in primary cortical astrocytes (**A**) and Iba1 (green) and CD68 (red) expression in primary cortical microglia (**B**) after 1, 2 or 3 days of treatment with media only (control), 100 pM $A\beta_{1-42}$, 100 pM N-Aβ fragment, 100 pM N- Aβcore, 100 pM $A\beta_{1-42}$ + 100 pM N-Aβ fragment and 100 pM $A\beta_{1-42}$ + 100 pM N-Aβcore. *Inset*: Magnified image of a typical GFAP-labeled astrocyte in A. and a typical Iba1-labeled microglia in B. Images obtained on a Leica TCS SP8 confocal microscope. Scale bar: 100μM. Primary antibodies [1:500 Mouse anti-GFAP antibody (Abcam) or 1:200 Rabbit anti-Iba1 antibody (Abcam) and 1:500 Rat anti-CD68 antibody (Biolegend)] were omitted in the secondary only samples. **C.** The percentage of primary cortical astrocytes with a high level of GFAP expression or **D.** primary cortical microglia with a high level of Iba1 and CD68 expression, after Aβ treatment as described above. (n=3), where n represents the number of independent experiments. Data are means ± SD.

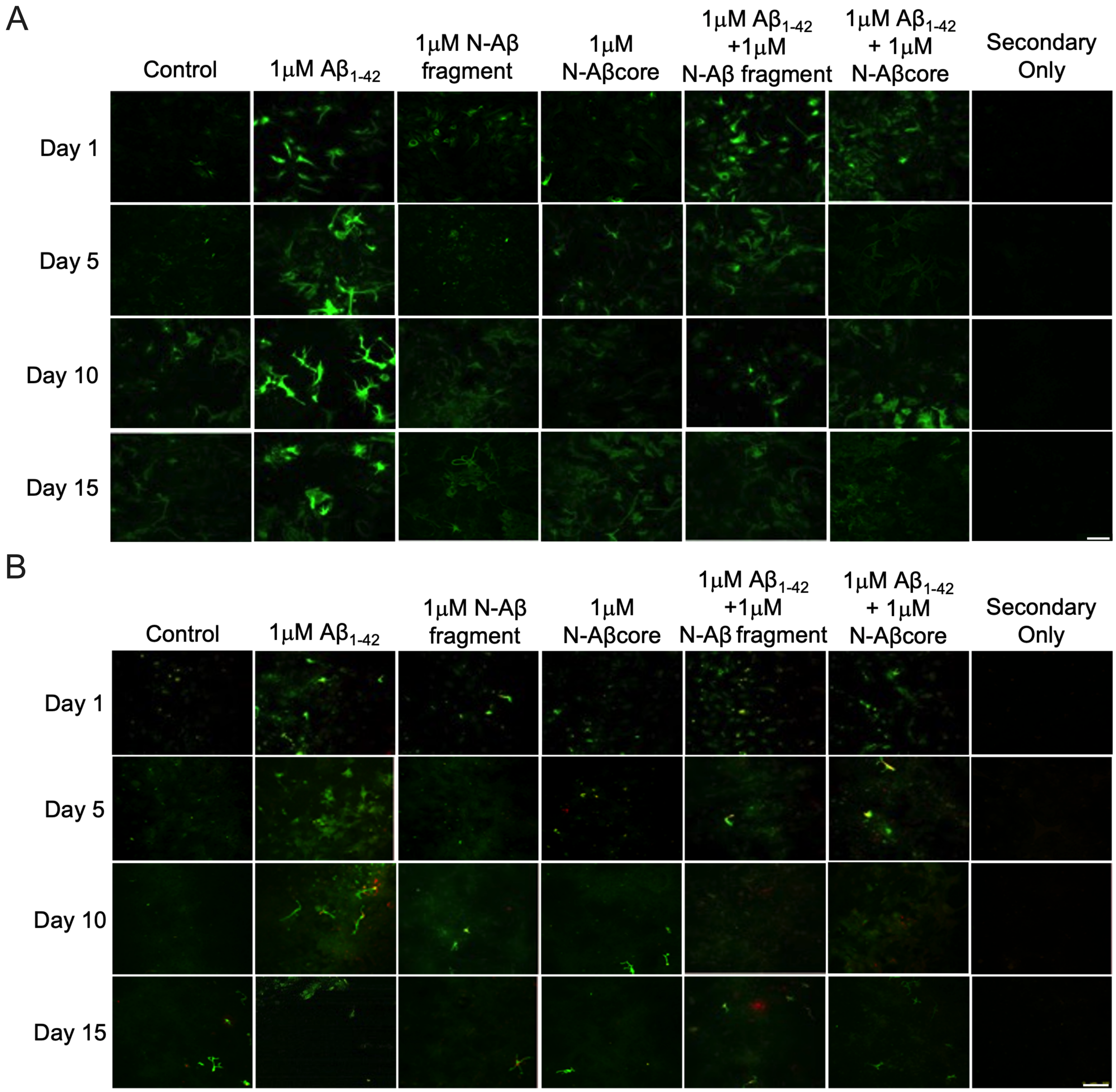

Fig. S5: The N-Aβ fragment and N-Aβcore mitigate the upregulation of GFAP expression in astrocytes and Iba1/CD68 expression in microglia induced by 1 μM $A\beta_{1\text{-}42}$

Representative images of GFAP expression in primary cortical astrocytes (**A**) or Iba1 (green) and CD68 (red) expression in primary cortical microglia (**B**) after 1, 5, 10 or 15 days of daily treatment with media only (Control), 1 μM $A\beta_{1\text{-}42}$, 1 μM N-Aβ fragment, 1 μM Aβcore, 1 μM $A\beta_{1\text{-}42}$ + 1 μM N-Aβ fragment, or 1 μM $A\beta_{1\text{-}42}$ + 1 μM N-Aβcore. Images obtained on a Leica TCS SP8 confocal

microscope. Scale bar: 100μM. Primary antibodies [1:500 Mouse anti-GFAP antibody (Abcam) or 1:200 Rabbit anti-Iba1 antibody (Abcam) and 1:500 Rat anti-CD68 antibody (Biolegend)] were omitted in the secondary only samples. (*n*=3 per treatment per treatment day), where *n* represents the number of independent experiments.

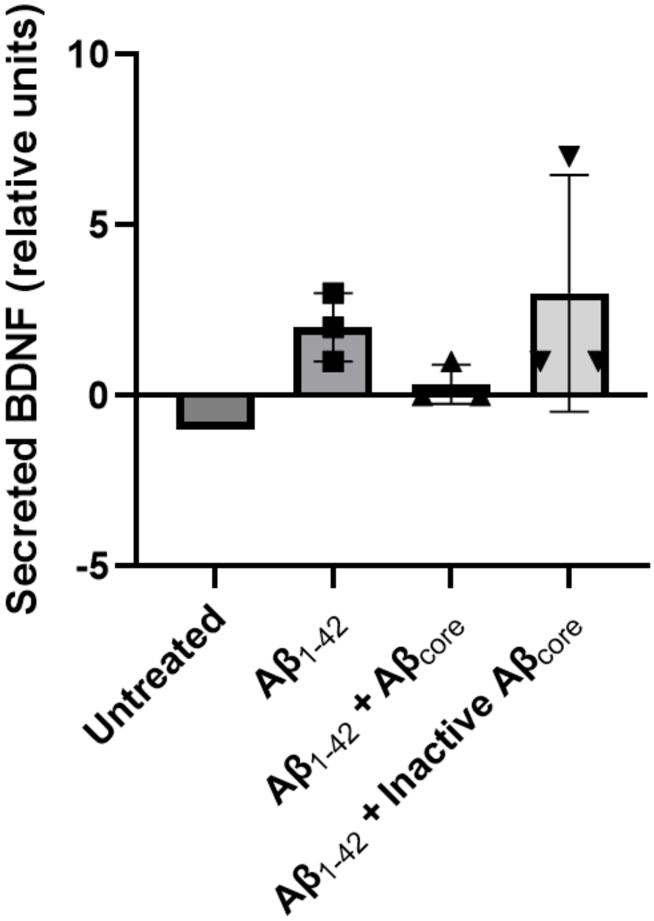

Fig. S6: N-Aβcore co-treatment reverses $A\beta_{1-42}$-induced secretion of the neurotrophin BDNF in BV2 cells

BV2 cells were treated as described for Figure 3. BDNF present in culture supernatants, representing secreted protein normalized to total supernatant protein, was assessed for each condition using a dot-blot immunoassay, as described in Methods.

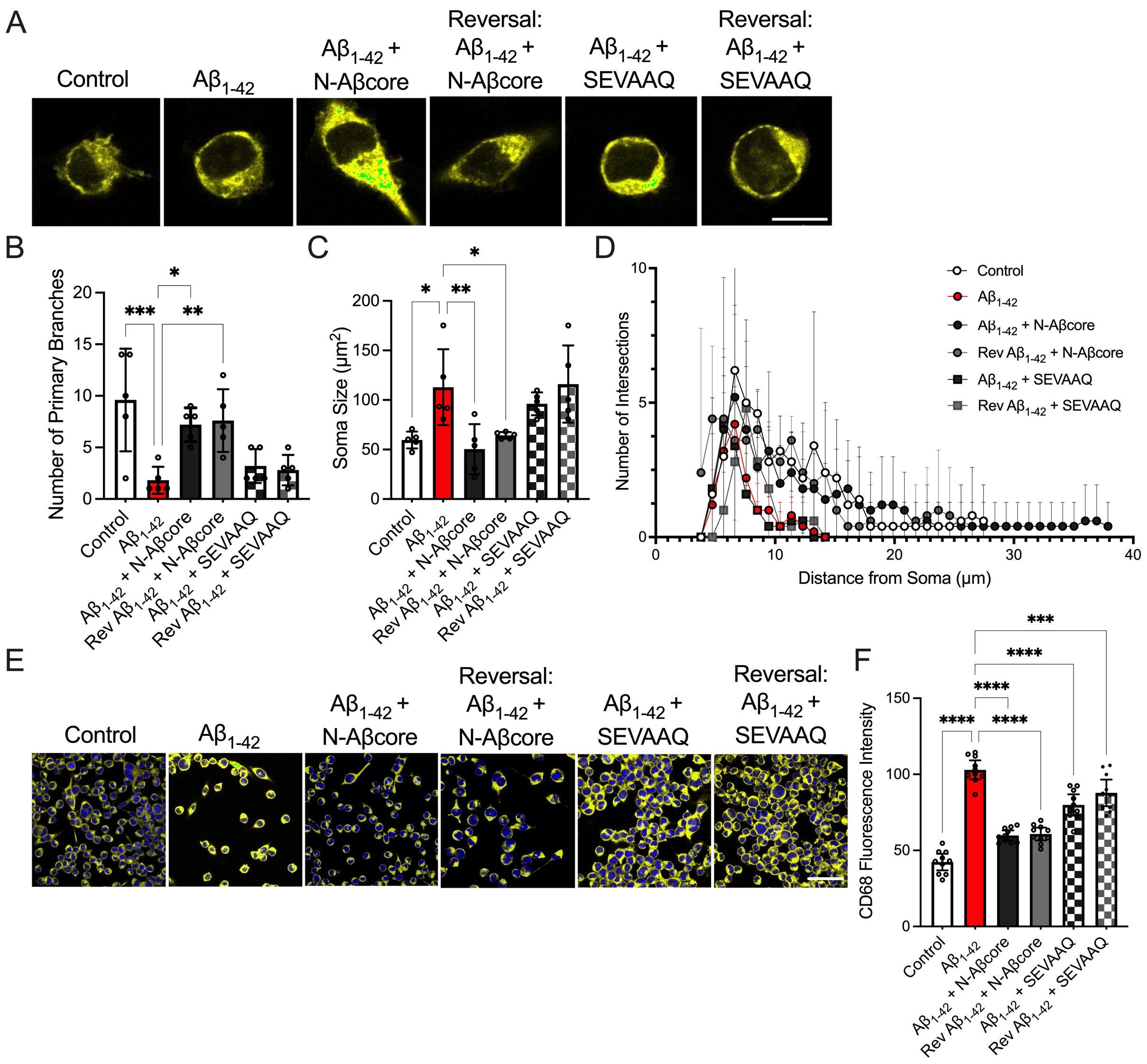

Fig. S7: N-Aβcore co-treatment attenuates $A\beta_{1\text{-}42}$-induced morphological changes and increased expression of CD68 in BV2 cells

BV2 cells were treated daily for 5 days with media only (Control), 500 nM $A\beta_{1\text{-}42}$, 500 nM $A\beta_{1\text{-}42}$ + 500 nM N-Aβcore or 500 nM $A\beta_{1\text{-}42}$ + 500 nM SEVAAQ (inactive substituted N-Aβcore). Cells were treated daily for 6 days with 500 nM $A\beta_{1\text{-}42}$ in the reversal conditions. **A**. Representative images of CD68-positive BV2 cells. Scale bar: 10 μm. Quantification of the number of primary branches (**B**) and soma size (**C**) of individual BV2 microglial cells (*n*=5). **D**. Sholl analysis indicating the number of microglial processes intersecting concentric circles drawn at 1 μm-step sizes from the center of the cell. **E.** Representative images of CD68-positive BV2 cells counterstained with DAPI. Scale bar: 50 μm. **F.** Quantification of CD68 expression in E. Images in A. and E. obtained using

a Leica TCS SP8 confocal microscope. Data are means ± SD. All data analyzed via one-way ANOVA with Dunnett *post hoc* test as compared to Aβ_{1-42}. * $p<0.05$ ** $p<0.01$ *** $p<0.001$ **** $p<0.0001$

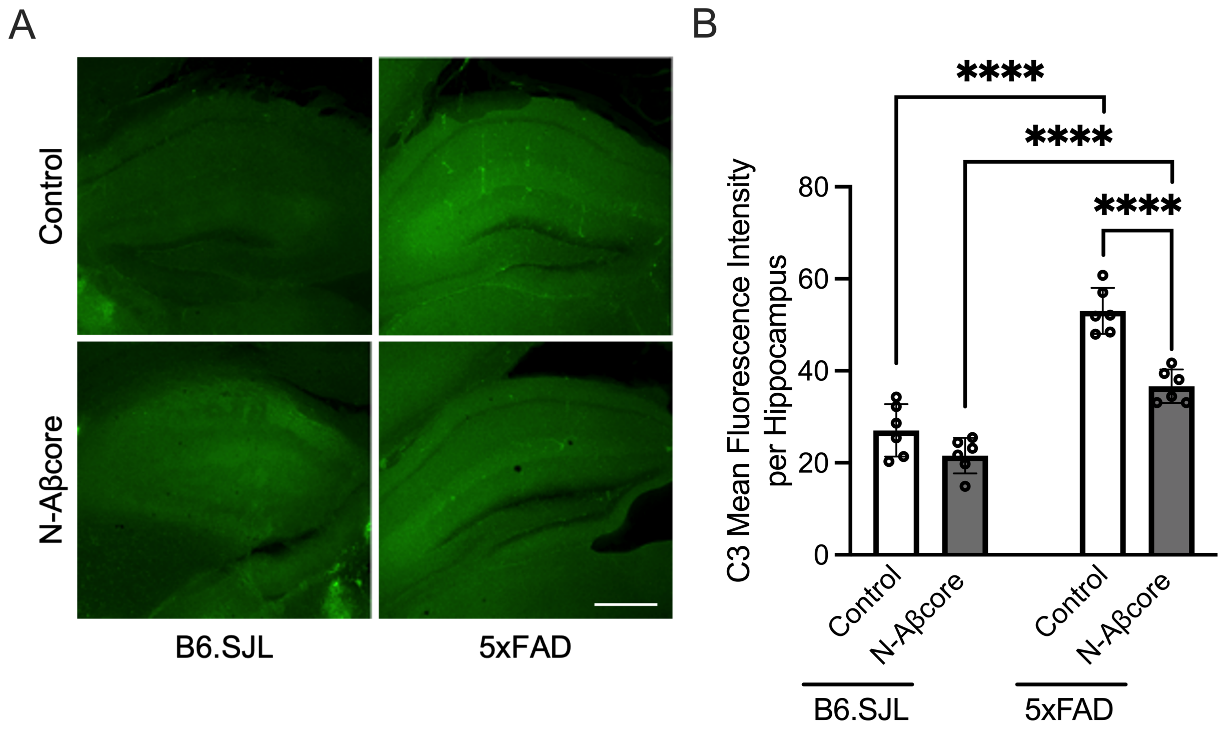

Figure S8: N-Aβcore mitigates complement C3 expression in organotypic slice cultures from 5xFAD mice

A. Representative images of complement C3 (green) labeled 3-month-old B6.SJL or 5xFAD organotypic slice cultures after 7 days daily treatment with media only (Control) or 1 µM N-Aβcore. Images obtained on a Leica SP8 confocal microscope using a 5X objective. Scale bar: 500 µm. **B.** Quantification of hippocampal C3 expression after treatments as described in A. (*n*=6 replicates per treatment group from 3 mice). 1 cultured slice = 1 replicate. Data are means ± SD, analyzed via one-way ANOVA with Dunnett *post hoc* test.

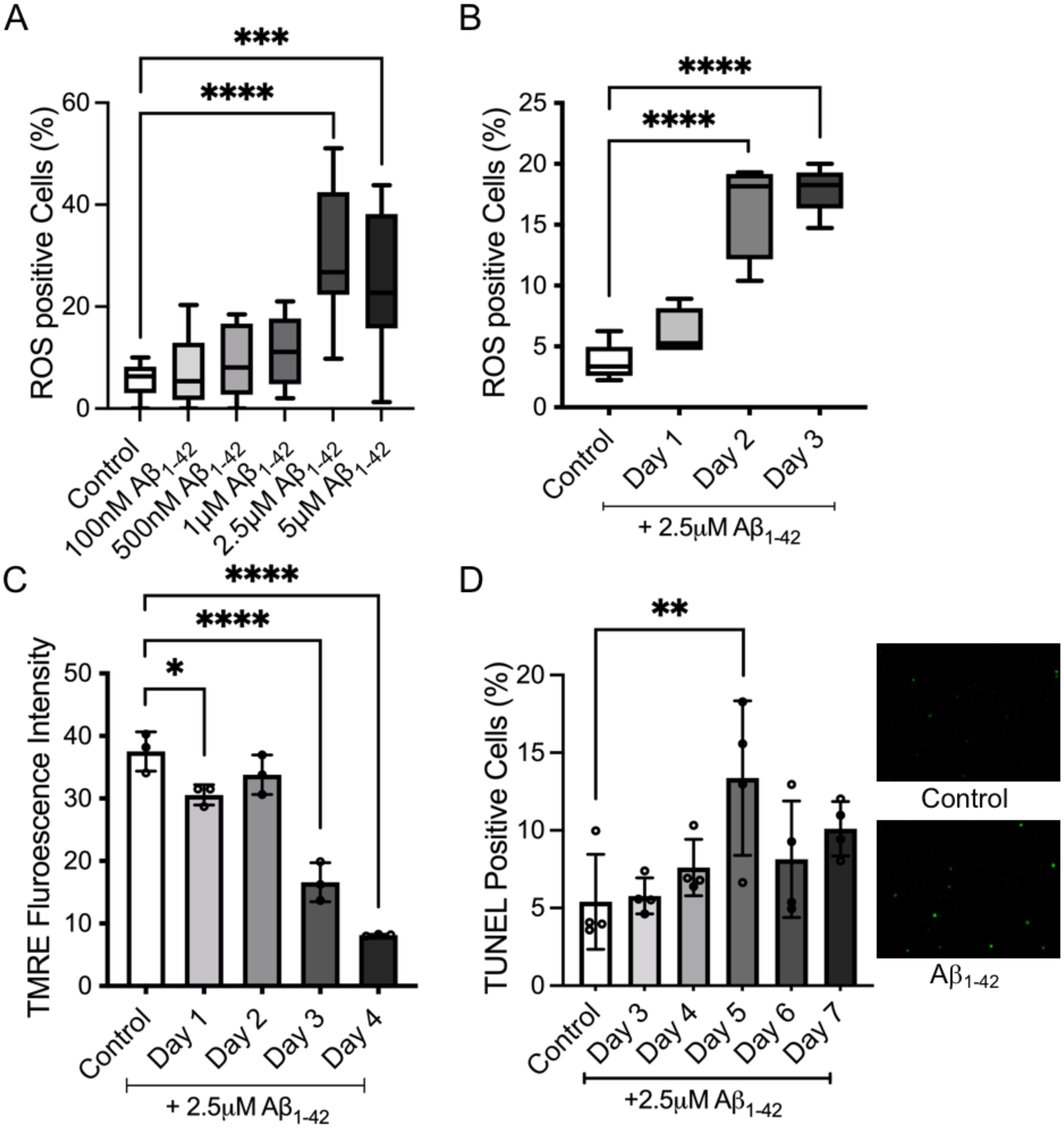

Fig. S9: Treatment with A$\beta_{1\text{-}42}$ induces oxidative stress, mitochondrial dysfunction and apoptosis in primary cortical astrocytes and microglia in a dose- and time-dependent manner

A. Percentage of ROS-positive astrocytes and microglia after daily treatment for 3 days with media only (Control), 100 nM A$\beta_{1\text{-}42}$, 500 nM A$\beta_{1\text{-}42}$, 1 μM A$\beta_{1\text{-}42}$, or 2.5 μM A$\beta_{1\text{-}42}$. (*n*=3). **B**. Percentage of ROS-positive astrocytes and microglia after daily treatment for 3 days with media

only (Control) or 1-3 days with 2.5 μM $A\beta_{1-42}$ (*n*=3). **C.** Mitochondrial membrane potential disruption was quantified as normalized values ($\Delta F/F_0$) for TMRE staining in individual astrocytes and microglia after daily treatment for 4 days with media only (Control) or 1-4 days with 2.5 μM $A\beta_{1-42}$ (*n*=3 individual experiments). **D.** Left: Percentage of TUNEL-positive astrocytes and microglia after daily treatment for 7 days with media only (Control) or 3-7 days with 2.5 μM $A\beta_{1-42}$ (*n*=3). Right: Representative images of TUNEL staining in Control and Day 5 $A\beta_{1-42}$ treated cells. All images were obtained on an Olympus IX71 fluorescent microscope via a 40X objective. Oxidative stress and apoptosis were quantified as the percent of mean cell counts per experimental *n* (total number of independent experiments). Data in A. and B. are represented as a box-and-whisker plots across 5-95 percentile range, with the lines indicating median values. Averaged data in C. and D. are means ± SD. (Dunnett *post hoc* test as compared to Control) * $p<0.05$ ** $p<0.01$ *** $p<0.001$ **** $p<0.0001$

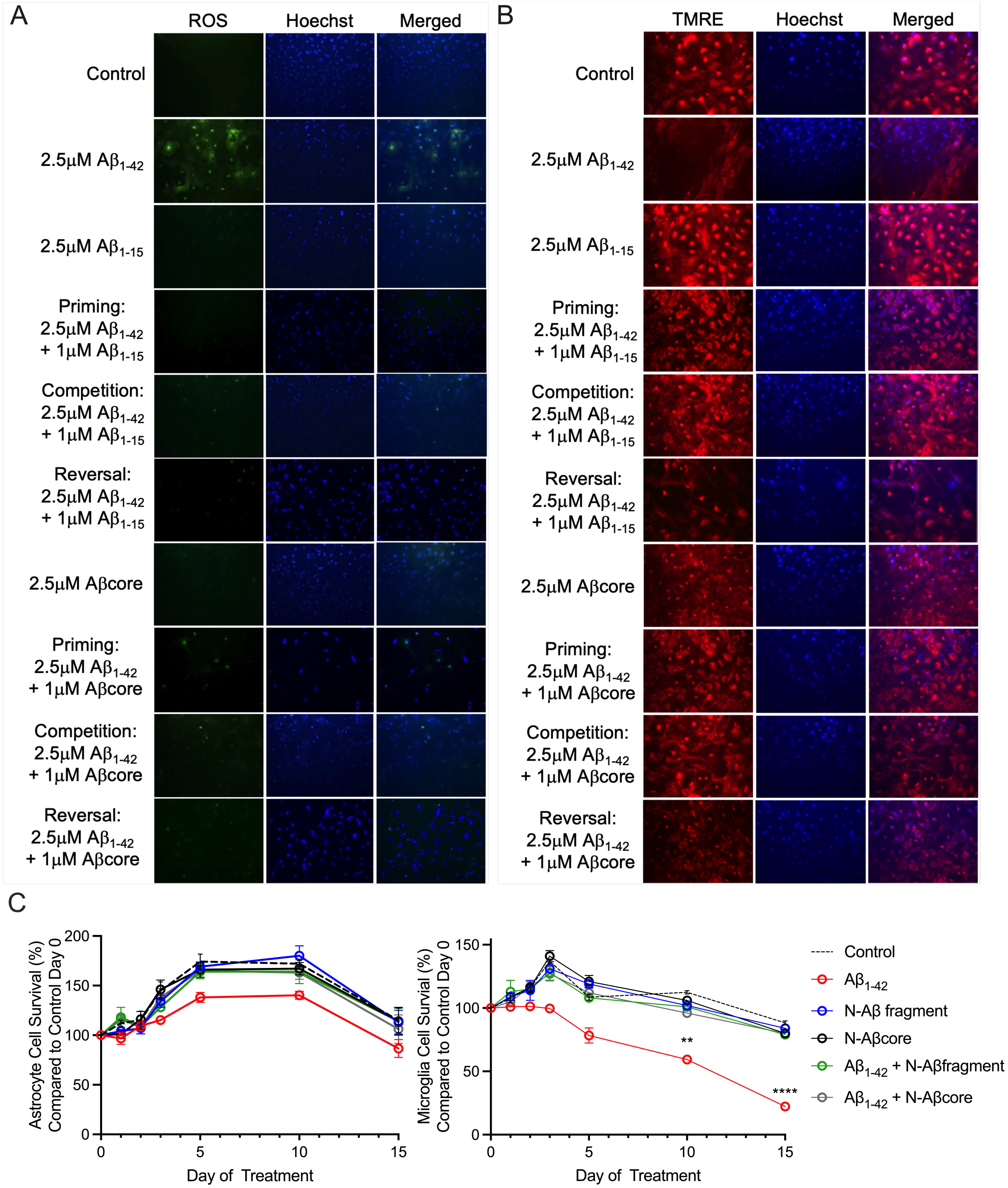

Figure S10: N-Aβ fragment and N-Aβcore mitigate $Aβ_{1-42}$-induced oxidative stress, mitochondrial dysfunction and cellular death in primary cortical astrocytes and microglia

Primary cortical astrocytes and microglia from mixed glial cultures treated daily with media only (Control), 2.5 μM Aβ_{1-42}, 2.5 μM N-Aβ fragment, 2.5 μM N-Aβcore, 2.5 μM Aβ_{1-42} + 1 μM NAβ fragment or 2.5 μM Aβ_{1-42} + 1 μM N-Aβcore under priming, competition or reversal conditions (see diagram in Fig. 6A). Representative images of ROS staining (**A**) and TMRE staining (**B**) in astrocytes and microglia. Nuclei were labelled with Hoechst 33342. All images were obtained on an Olympus IX71 fluorescent microscope via a 40X objective. **C**. Percent cell survival compared to Control Day 0 as determined by direct cell counts of primary cortical astrocytes (left) and microglia (right) after 1, 5, 10 or 15 days of daily treatment (*n*=3), where *n* is the total number of experiments. Data analyzed via one-way ANOVA with Dunnett *post hoc* test as compared to untreated Control for each treatment day. ** $p<0.01$ **** $p<0.0001$

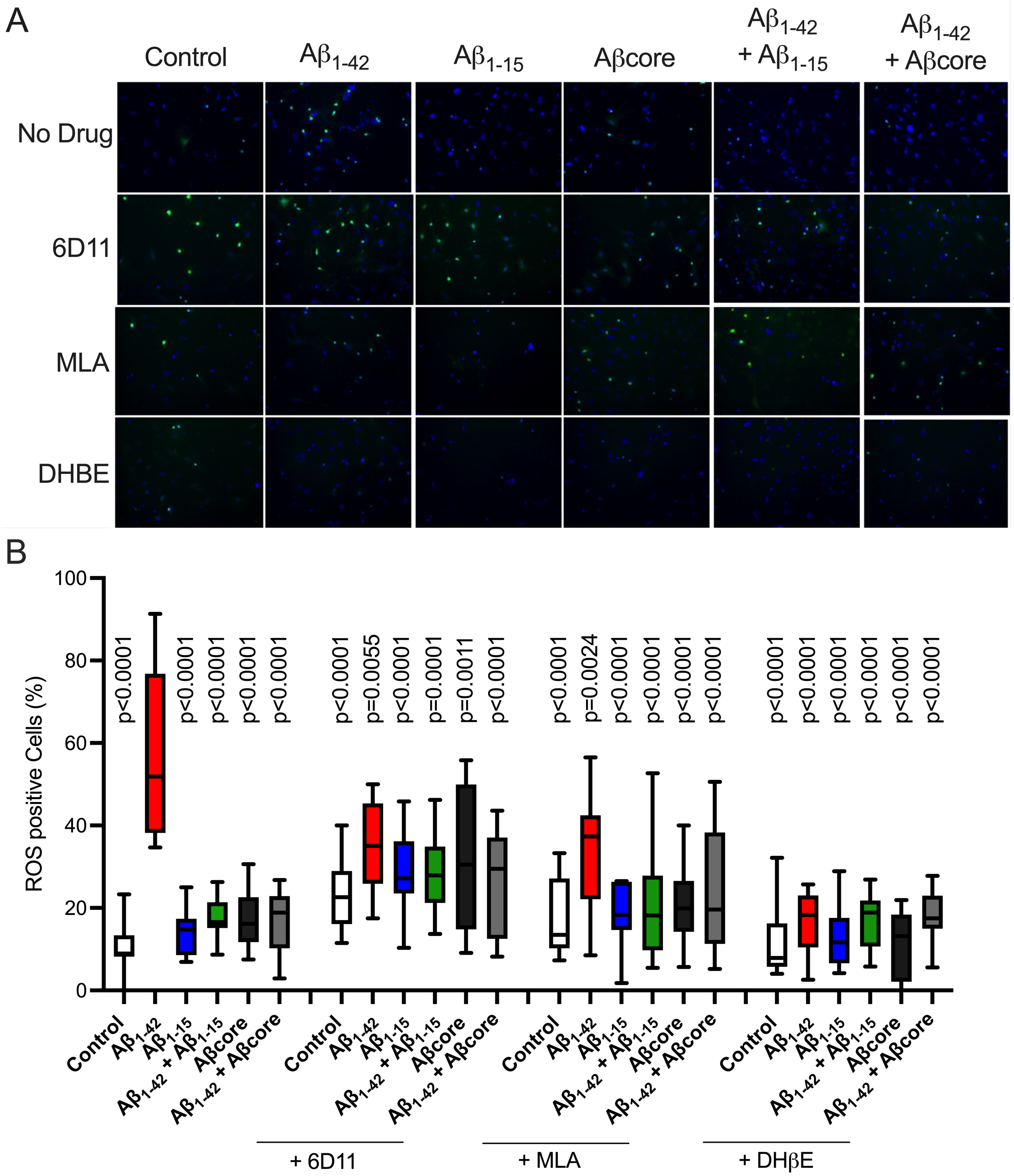

Figure S11: Selective antagonists of PrP^c, α7-nAChR or α4-nAChR pathways partially compromise the protective functions of the N-Aβ fragment or N-Aβcore

against the oxidative stress induced by full-length $A\beta_{1-42}$ in primary cortical astrocytes and microglia

A. Representative images of ROS staining. Nuclei were labelled with Hoechst 33342. All images were obtained on an Olympus IX71 fluorescent microscope via a 40X objective. **B**. Quantification of oxidative stress as the percent of mean counts of ROS-positive cells per experimental *n* (total number of independent experiments). Glial cultures were incubated with media only (no drug), anti-PrP^c antibody 6D11, 10 nM MLA (methyllycaconitine) or 100 nM DHBE (dihydro-β-erythroidine) for 3 hours prior to treatment with media only (Control), 2.5 μM $A\beta_{1-42}$, 2.5 μM $A\beta_{1-15}$, 2.5 μM Aβcore, 2.5 μM $A\beta_{1-42}$ + 1 μM $A\beta_{1-15}$ or 2.5 μM $A\beta_{1-42}$ + 1 μM Aβcore in media containing no drug, anti-PrP^c antibody 6D11, 10 nM MLA or 100 nM DHBE daily for 2 days. (*n*=3). Data are represented as a box-and-whisker plot across 5-95 percentile range, with the lines indicating median values. P values < 0.05 shown. All data analyzed via one-way ANOVA with Dunnett *post hoc* test as compared to 2.5 μM $A\beta_{1-42}$ from the No Drug group. N-Aβ fragment: $A\beta_{1-15}$.
